# Molecular Insights into HPV-Driven Head and Neck Cancers: From Viral Oncoproteins to Precision Therapeutics

**DOI:** 10.3390/v17091276

**Published:** 2025-09-20

**Authors:** Mustafa Ozdogan, Gizem Tutkun, Muharrem Okan Cakir, Gholam Hossein Ashrafi

**Affiliations:** 1 Division of Medical Oncology, Memorial Hospital, Antalya 07050, Turkey; 2 Department of Medical of Biotechnology, Institute of Health Sciences, Akdeniz University, Antalya 07058, Turkey; gizemtutkun17@gmail.com; 3 School of Life Sciences, Pharmacy and Chemistry, Kingston University London, London KT1 2EE, UK; m.okan@kingston.ac.uk (M.O.C.); h.ashrafi@kingston.ac.uk (G.H.A.)

**Keywords:** human papillomavirus, HPV, head and neck squamous cell carcinoma, HNSCC, E6/E7 oncoproteins, biomarkers

## Abstract

Human papillomavirus (HPV) plays a major role in the development of head and neck cancers (HNCs), particularly oropharyngeal squamous cell carcinoma. This review highlights the key molecular mechanisms of HPV-driven carcinogenesis, focusing on the oncogenic E6 and E7 proteins and their disruption of tumor suppressor pathways and epigenetic regulation. We discuss the rising prevalence of HPV-related HNCs, their distinct clinical features, and diagnostic approaches such as p16 immunohistochemistry and HPV DNA/RNA detection. HPV-positive tumors show better prognosis and response to treatment, prompting interest in therapy de-escalation. Emerging strategies including immune checkpoint inhibitors, therapeutic vaccines, CRISPR-based gene editing, and ctDNA monitoring are advancing precision oncology in this field. We also examine the preventive potential of HPV vaccination and ongoing research into its role across various HNC subtypes. A deeper understanding of HPV’s molecular impact may guide more effective, targeted, and less toxic interventions.

## 1. Introduction

Head and neck cancers (HNCs) rank among the top ten most common malignancies worldwide, affecting the upper respiratory and digestive tracts [1]. They encompass a variety of histological subtypes, the majority being squamous cell carcinomas [2]. Established risk factors include tobacco use, excessive alcohol consumption, and infection with human papillomavirus (HPV) [3]. In the United States and parts of Europe, 60–70% of newly diagnosed oropharyngeal squamous cell carcinomas (OPSCC) are now attributable to HPV [4,5].

Globally, HNCs account for approximately 3–5% of all cancers, with more than 660,000 new cases and 350,000 deaths annually [1]. The burden of HPV-related OPSCC is increasing rapidly, and projections suggest that by 2030 the majority of OPSCC cases will be HPV-associated [6]. Within the oropharynx, the palatine tonsils are the most frequently affected site, followed by the base of tongue and soft palate [7].

HPV is a DNA virus implicated in several cancers, including those of the cervix, anus, and oropharynx. Its oncogenic potential lies in high-risk types, particularly HPV16 and HPV18, which drive malignant transformation through expression of the viral oncoproteins E6 and E7 [8,9]. These proteins inactivate the tumor suppressors p53 and retinoblastoma protein (pRb), resulting in uncontrolled cell-cycle progression and malignant transformation [10].

Understanding the mechanisms by which HPV contributes to the pathogenesis of HNCs is essential for developing effective strategies for prevention, early detection, and treatment. This review provides a comprehensive overview of HPV-associated HNCs, addressing their epidemiology, molecular virology, clinical features, and therapeutic implications.

## 2. Epidemiology, Prevalence and Demographic Trends of HPV-Related HNCs

HNCs represent a major global health burden [11]. Historically, the main etiological factors were tobacco and alcohol consumption [3], but the rising contribution of HPV particularly in OPSCC has shifted the epidemiological profile of HNCs [12]. The epidemiological and anatomical distribution of HPV-related HNCs is summarized in Table 1.

**Figure 1:**
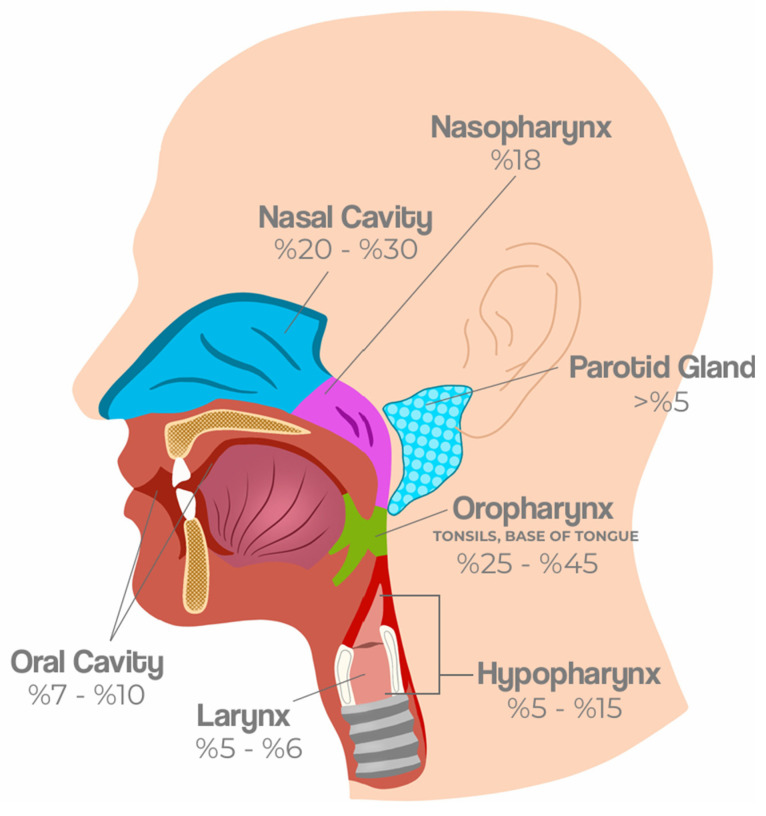
Anatomical distribution and prevalence of HPV-related HNCs. HPV-related HNCs most frequently arise in the oropharynx, particularly the tonsils and base of the tongue (25−45%). Other affected subsites include the oral cavity (7−10%), nasopharynx (18%), larynx (5−6%), hypopharynx (5–15%), nasal cavity (20–30%), and parotid gland (>5%). These patterns reflect both regional epidemiological variation and distinct biological predispositions of lymphoid-rich tissues to HPV-driven carcinogenesis [1,13,14,15].

Beyond clinicopathological differences, HPV-related HNCs show distinct anatomical patterns. The oropharynx, especially the tonsils and base of tongue, is the most frequent site, followed by less common subsites such as the hypopharynx, nasopharynx, sinonasal cavity, and salivary glands (Figure 1).

The incidence of HPV-positive OPSCC has risen sharply in North America and Europe, in contrast to HPV-negative disease which has declined due to reduced tobacco use. For example, between 1988 and 2004, HPV-positive OPSCC increased by 225%, whereas HPV-negative OPSCC decreased by 50% [16]. In contrast, in many Asian and African regions, HPV-negative tumors remain more prevalent [17].

HPV-positive HNCs are anatomically distinct, most frequently arising in lymphoid-rich areas of the oropharynx, especially the tonsils and base of tongue, with rarer cases in the hypopharynx, nasopharynx, and sinonasal tract [18]. These cancers are strongly associated with sexual behaviors, including a higher number of oral sex partners [14].

Demographically, HPV-positive HNCs differ from HPV-negative tumors. They are more common in younger to middle-aged patients, predominantly in men (male-to-female ratio ~3–4:1), and occur more often in Caucasian populations [19]. However, recent reports suggest a shifting age distribution, with increasing diagnoses in older cohorts, likely reflecting birth cohort effects [18].

HPV type distribution also differs by site. In OPSCC, HPV16 accounts for >80–90% of cases, HPV33 contributes ~3–5%, and HPV18 is rare (~2–3%) [20,21]. This contrasts with cervical cancer, where HPV16 and HPV18 together dominate the majority of cases.

Patients with HPV-positive OPSCC often present with small primary tumors but advanced cervical lymphadenopathy, usually as painless neck masses. Despite nodal burden, they generally show better response to chemoradiotherapy, and improved survival outcomes compared with HPV-negative patients [22].

**Table 1:** Comparative Clinical, Epidemiological, and Pathological Features of HPV-positive and HPV-negative Head and Neck Squamous Cell Carcinoma (HNSCC) [12,17,19].

Feature	HPV-Related HNC	Notes
**Common anatomical subsites**	Oropharynx (tonsils, base of tongue, soft palate, pharyngeal wall)	Tonsil is the most frequent site
**Less common subsites**	Larynx, hypopharynx, sinonasal cavity, nasopharynx, salivary glands	HPV prevalence varies by cohort, usually lower than oropharynx
**Histological type**	Predominantly squamous cell carcinoma (SCC)	Non-keratinizing basaloid morphology more frequent in HPV+ tumors
**Global incidence**	>660,000 new HNC cases annually; ~30–40% of OPSCC are HPV+ globally	Incidence rising in Europe and North America
**HPV type distribution**	HPV16 (~80–90%), HPV33 (~3–5%), HPV18 (~2–3%)	Differs from cervical cancer, where HPV16/18 dominate
**Demographic trends**	Higher incidence in men vs. women (3–4:1); increasing in older cohorts	Linked to sexual behavior and birth cohort effects
**Clinical presentation**	Often presents with small primary tumor and early lymph node metastasis	Painless cervical lymphadenopathy is common initial sign
**Distant Organ Metastasis**	Unusual sites: skin, brain	Lung
**Histopathological Features**	Non-keratinizing or basaloid	Keratinizing
**Tumor Differentiation**	Undifferentiated	Differentiated
**Sensitivity to Chemoradiotherapy**	Better response	Worse response
**Prognosis (Survival)**	Better prognosis	Worse prognosis

## 3. Virology and Mechanism of HPV in HNCs

**Figure 2:**
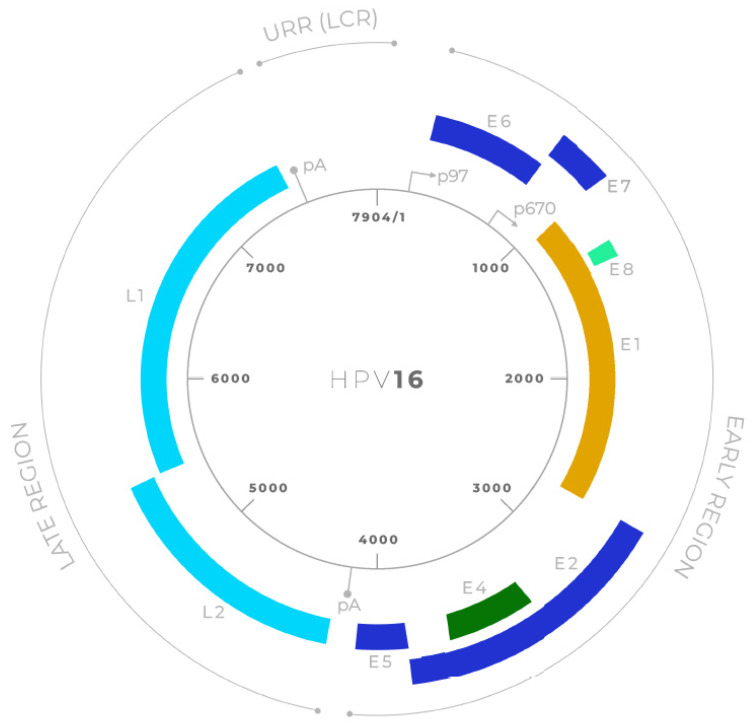
Schematic representation of the HPV16 genome organization [26]. The circular double-stranded DNA genome of Human Papillomavirus type 16 (HPV16; 7904 bp) is shown, divided into early (E) and late (L) regions. Early genes include E6, E7, E1, E2, E4, E5, and E8, which are involved in viral replication, transcriptional regulation, and oncogenesis. Late genes L1 and L2 encode the major and minor capsid proteins, respectively. The upstream regulatory region (URR; also known as the long control region, LCR) contains the viral origin of replication and transcriptional control elements. Major promoters (p97, p670) and polyadenylation sites (pA) are indicated.

HPVs are non-enveloped viruses belonging to the *Papillomaviridae* family [23]. They possess a circular double-stranded DNA genome of approximately 8000 base pairs [24]. The HPV genome is divided into three main regions (Figure 2): the early (E) region, the late (L) region, and the long control region (LCR) or upstream regulatory region (URR). The early region encodes proteins essential for viral replication and transcription regulation, including E1, E2, E4, E5, E6, E7 and E8. The late region encodes the structural proteins L1 and L2, which form the viral capsid [25].

HPV is a central factor in the etiology of various malignancies, including HNCs. Its oncogenic potential stems from the ability of viral proteins to disrupt key tumor suppressor pathways within host cells [27].

### 3.1. Oncogenic Mechanism of HPV

HPVs are divided into low-risk and high-risk types based on their oncogenic potential. Low-risk types, such as HPV6 and HPV11, are associated mainly with benign conditions including genital warts and recurrent respiratory papillomatosis, and they rarely progress to malignancy [28]. In contrast, high-risk types such as HPV16, 18, 26, 31, 33, 35, 39, 45, 51, 52, 53, 56, 58, 59, 66, 68, 70, 73, and 82 account for the vast majority of HPV-related cancers worldwide. These genotypes are responsible for over 90% of cervical cancers, most anal cancers, and a substantial proportion of vaginal, vulvar, penile, and HNCs [29].

A key distinction between low-risk and high-risk HPVs is their capacity to integrate into the host genome and the oncogenic consequences that follow. In benign lesions, such as genital warts, HPV typically persists in an episomal form, separate from host chromatin. By contrast, viral integration frequently in cervical carcinogenesis and present in a subset of HPV-positive HNSCCs is a critical driver of malignant progression. Integration disrupts the viral E2 gene, leading to uncontrolled expression of the viral oncogenes E6 and E7, while also contributing to host genomic instability [30,31]. However, not all HPV-positive HNSCCs are driven by integrated genomes; many tumors maintain an episomal form, or a mixture of episomal and integrated genomes. In such cases, viral gene expression is controlled by epigenetic and transcriptional mechanisms such as YY1/CTCF chromatin architecture and SETD2-dependent histone methylation [32,33,34]. Clinical studies suggest that integrated status may be linked with more aggressive tumor biology in a subset of patients, highlighting the potential utility of viral genome status as a biomarker for stratifying patients and guiding treatment strategies [30,31,33].

Beyond integration, viral proteins play central roles in oncogenesis. The E5 protein localizes to the endoplasmic reticulum and Golgi apparatus, where it interferes with antigen presentation by reducing the transport of MHC class I molecules to the cell surface. This enables immune evasion by limiting recognition of infected cells by cytotoxic T lymphocytes, thereby supporting viral persistence and potentially contributing to carcinogenesis [35].

**Figure 3:**
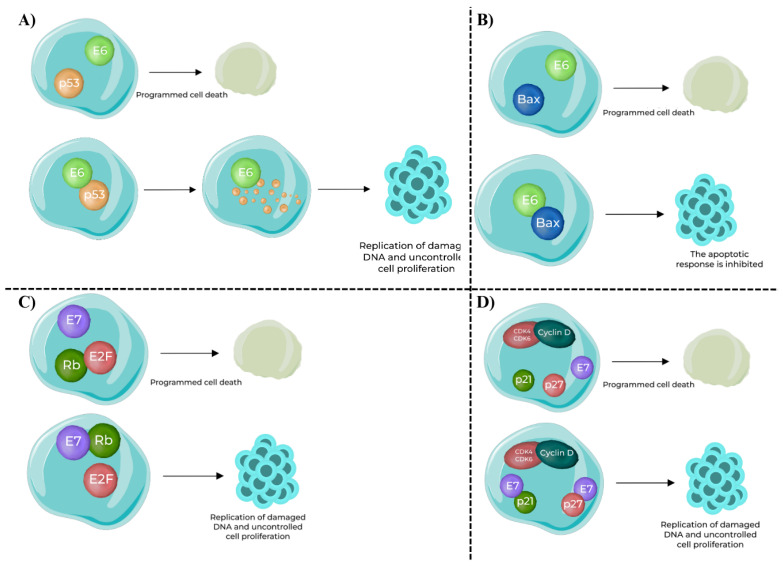
Oncogenic functions of HPV E6 and E7 oncoproteins in host cells [45]. (**A**) E6–p53 interaction: In normal conditions, p53 induces programmed cell death in response to DNA damage. HPV E6 binds to p53 and promotes its ubiquitination and degradation, preventing apoptosis and enabling replication of damaged DNA. (**B**) E6–Bax interaction: Bax, a pro-apoptotic protein, normally promotes apoptosis. Binding of E6 to Bax inhibits its function, blocking the apoptotic response and supporting uncontrolled cell survival. (**C**) E7–Rb interaction: Retinoblastoma protein (Rb) sequesters E2F transcription factors to prevent inappropriate cell cycle progression. HPV E7 binding inactivates Rb, releasing E2F and driving expression of S-phase genes, resulting in uncontrolled proliferation. (**D**) E7–CDK inhibitors interaction: Cyclin-dependent kinase inhibitors p21 and p27 normally block the Cyclin D–CDK4/6 complex, enforcing the G1/S checkpoint. HPV E7 inhibits p21 and p27, removing this control and further accelerating cell cycle progression and replication of damaged DNA.

The oncogenic potential of high-risk HPV types is, however, largely mediated by E6 and E7 oncoproteins (Figure 3). E6 binds to and promotes the degradation of the tumor suppressor protein p53, thereby eliminating a key regulator of DNA repair and apoptosis. E6 also targets the pro-apoptotic protein Bax, further suppressing programmed cell death and allowing damaged cells to survive [36,37,38]. E6 also induces expression of the catalytic subunit of telomerase (hTERT), leading to telomere maintenance and cellular immortalization, a critical step in malignant transformation [39,40]. In parallel, E7 binds to the retinoblastoma protein (pRb), disrupting its ability to sequester E2F transcription factors. Beyond functional inactivation, E7 promotes proteasome-mediated degradation of pRB, thereby ensuring persistent E2F release and uncontrolled entry into S phase [41,42]. E7 also inhibits cyclin-dependent kinase inhibitors such as p21 and p27, enhancing cyclin D–CDK4 activity and driving unchecked proliferation [43,44].

In conclusion, the oncogenic mechanisms of HPV involve a multifaceted interplay between viral genome status and viral oncoproteins. Integration events can deregulate E6/E7 expression, while E5 facilitates immune evasion. Together, the actions of E5, E6, and E7 converge on key tumor suppressor pathways, particularly those mediated by p53 and Rb, driving uncontrolled proliferation and malignant transformation. Elucidating these mechanisms is essential for developing preventive and therapeutic strategies against HPV-related HNCs [46,47].

### 3.2. HPV Mechanisms Affected by Epigenetic Regulation

**Figure 4:**
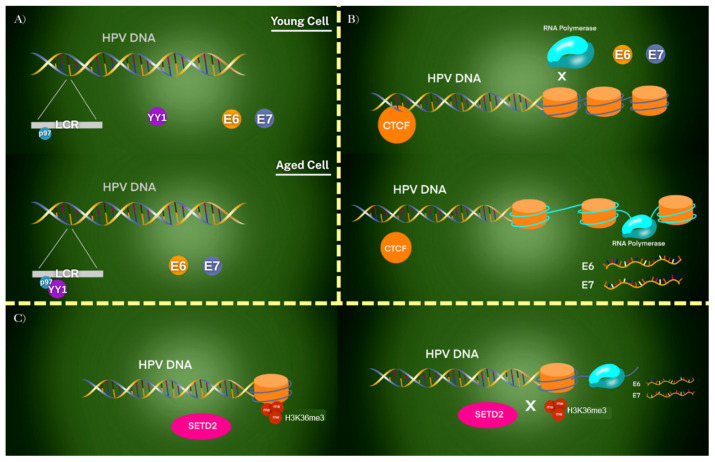
Epigenetic regulation of HPV oncogene expression in different chromatin contexts. (**A**) In young undifferentiated epithelial cells, YY1 binds to the LCR and represses the p97 promoter, silencing E6 and E7 oncogenes. With cellular aging or differentiation, YY1 binding is lost, leading to derepression and activation of E6/E7. (**B**) CTCF establishes a repressive chromatin loop in undifferentiated cells, preventing RNA polymerase II access to the promoter. As cells differentiate, this loop is disrupted, RNA polymerase gains access, and viral oncogenes are transcribed. Host proteins such as SIRT1 and WRN further modulate this process by regulating replication and chromatin state. (**C**) SETD2 introduces H3K36me3 epigenetic marks on viral chromatin. While these marks enhance transcription of certain viral genes, they are absent from E6/E7 loci, allowing these oncogenes to escape repression and remain transcriptionally active. Together, these mechanisms demonstrate how loss of epigenetic control leads to persistent E6/E7 expression and malignant transformation.

The activity of HPV oncogenes is strongly influenced by epigenetic regulation, which involves chemical and structural modifications of DNA and chromatin. The p97 promoter, located within the long control region (LCR), serves as a key regulatory hub. Its activity is modulated by epigenetic marks and transcription factors that either suppress or activate viral oncogene expression [47]. For example, the transcription factor Yin Yang 1 (YY1) plays a central role in repressing E6 and E7 expression in undifferentiated epithelial cells. As epithelial tissues mature, YY1 binding is progressively lost, leading to derepression of the p97 promoter and activation of E6/E7 transcription (Figure 4A) [22].

In addition to YY1, the chromatin organizer CCCTC-binding factor (CTCF) contributes to epigenetic control by forming repressive chromatin loops within the HPV genome. These loops prevent access of RNA polymerase and restrict viral oncogene transcription. During differentiation, the disruption of these loops enables E6 and E7 expression [32,48]. Other host proteins, including Sirtuin1 (SIRT1) and Werner Syndrome Protein (WRN), also participate in regulating HPV replication and epigenetic modifications of the viral genome, further modulating viral gene activity and oncogenic potential (Figure 4B) [49].

A further layer of regulation involves histone methylation. HPV31 E7 has been shown to upregulate SETD2, a histone methyltransferase responsible for depositing H3K36me3 marks on viral chromatin. This modification promotes transcription of selected viral genes. Importantly, E6 and E7 loci lack H3K36me3 deposition, allowing these oncogenes to evade repression and remain highly expressed (Figure 4C) [33,34]. Many foundational studies on YY1/CTCF regulation and SETD2-dependent histone methylation have been conducted in episomal HPV models. Thus, extrapolation to tumors with integrated HPV genomes requires caution, as differences in genome architecture may significantly influence oncogene expression and patient outcomes [32,33,34].

These epigenetic mechanisms play a key role in determining the extent of HPV’s pathogenicity within the host, contributing to its potential to induce malignancy.

### 3.3. The Role of DNA Methylation in the HPV Mechanism

**Figure 5:**
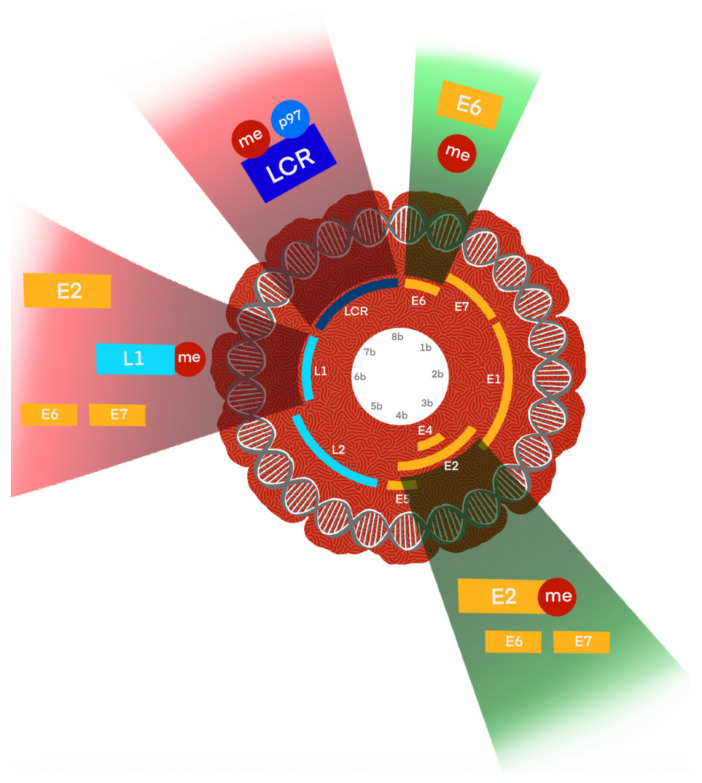
Schematic illustration of DNA methylation-mediated regulation of HPV genome regions and its oncogenic consequences. The HPV genome is shown in its circular episomal form, with key regulatory and coding regions indicated, including the long control region (LCR), early genes (E6, E7, E1, E2, E4, E5), and late genes (L1, L2). DNA methylation (me) at distinct loci has functional consequences: LCR and E2 binding sites (E2BS): Hypomethylated in low-grade lesions but hypermethylated in high-grade lesions, leading to deregulation of E6/E7 oncogene expression and promoting viral integration; E6 region: Methylation here can suppress oncogene transcription; loss of control contributes to carcinogenesis, while micronutrient-enhanced methylation (e.g., folate, vitamin B12) may provide protective effects; L1 region: Hypermethylation correlates with high-grade intraepithelial neoplasia and invasive cancers, serving as a biomarker of advanced disease. These methylation changes collectively modulate transcriptional activity, influence integration status, and ultimately drive malignant transformation in HPV-related cancers.

HPV’s DNA methylation patterns play a critical role in regulating viral replication, gene expression, and oncogenic transformation (Figure 5) [50]. These epigenetic alterations vary depending on the viral genotype and integration status, and they strongly influence disease progression.

In HPV16, E2 binding sites (E2BS) typically remain hypomethylated in low-grade lesions, maintaining normal viral transcriptional control. However, in high-grade lesions these sites become hypermethylated, correlating with activation of the early promoter and an increased likelihood of viral integration. Such methylation changes are critical events in the transition from benign to malignant disease [51].

Hypermethylation of the L1 region has also been consistently associated with high-grade lesions and invasive cervical cancer. These findings indicate that methylation at specific genomic sites can serve as a biomarker of disease severity and risk of progression [51]

HPV18 demonstrates a higher integration frequency than HPV16; nevertheless, viral integration is not always necessary for malignant transformation. Instead, abnormal DNA methylation at E2BS can disrupt the regulation of E6/E7 oncogenes independently of integration, thereby contributing to oncogenic progression [52].

Interestingly, environmental and nutritional factors also appear to influence methylation. Micronutrients such as folate and vitamin B12 have been shown to enhance methylation at the E6 region, which may provide a protective effect against the progression of low-grade CIN2+ lesions [47,53]. These findings suggest that methylation is not only a viral- and genotype-dependent process but also influenced by host factors.

These findings suggest that HPV methylation profiles are not only critical in regulating viral replication and gene expression but also play a key role in determining cancer risk. valuable insights for the development of diagnostic and therapeutic approaches.

### 3.4. Long Non-Coding RNAs

HPV alters host and viral non-coding RNA networks, which play a key role in malignant transformation. Non-coding RNAs (ncRNAs) include microRNAs (miRNAs), circular RNAs (circRNAs), and long non-coding RNAs (lncRNAs). Among these, lncRNAs—defined as transcripts longer than 200 nucleotides that lack protein-coding capacity—are particularly implicated in HNCs, as they regulate transcription, translation, and chromatin remodeling [54,55].

One example is CCEPR (cervical carcinoma expressed PCNA regulatory lncRNA), which localizes to both the nucleus and cytoplasm of oral squamous cell carcinoma cells. Acting as an oncogene, it sponges miR-922 and thereby increases expression of PAK2, a kinase involved in PI3K/AKT and MAPK/ERK signaling, as well as cytoskeletal dynamics, proliferation, and survival [56].

Another lncRNA, FAM83H-AS1, is highly expressed in cervical cancers and HPV16-positive HNSCC cell lines compared to HPV-negative counterparts. Its upregulation is driven by E6/E7 in a p53-independent but p300-dependent manner. Suppression of FAM83H-AS1 reduces proliferation, migration, and survival, underscoring its role in early carcinogenesis [57].

Conversely, PRINS (psoriasis susceptibility-related RNA gene induced by stress) is overexpressed in HPV-positive HNSCC and correlates with antiviral and immune response genes. Its expression promotes immune infiltration, which may help explain the better treatment responses observed in HPV-positive patients [58].

Additional dysregulated lncRNAs in HPV-positive HNSCC include CDKN2B-AS1, TTTY14, TTTY15, MEG3, and H19, many of which are associated with immune modulation and tumor suppression [47,59]. Collectively, these lncRNAs illustrate how HPV-driven transcriptomic reprogramming contributes to oncogenesis and highlight their potential as prognostic or therapeutic biomarkers.

### 3.5. Epigenetic Alterations and Their Potential as Biomarker

Distinct methylation and chromatin profiles have been identified in HPV-positive cancers, distinguishing them from both normal tissues and HPV-negative counterparts [47,60]. These epigenetic signatures correlate closely with disease stage, tumor aggressiveness, and survival outcomes, providing valuable insights into cancer progression. They also hold potential as biomarkers for diagnosis, prognosis, and recurrence prediction. Moreover, differences in these signatures across HPV genotypes contribute to variations in tumor biology and clinical behavior [60,61].

Emerging evidence suggests that epigenetic signatures may outperform traditional biomarkers such as p16 in sensitivity and specificity. Integrating epigenetic profiling into clinical practice could facilitate personalized treatment strategies and enable more precise patient risk stratification.

## 4. Clinical Presentation and Diagnosis

### 4.1. Symptoms and Presentation

HNCs present with diverse clinical features depending on their etiology, with HPV-related and HPV-negative tumors showing distinct characteristics [18]. HPV-related HNCs, which arise predominantly in the oropharyngeal region, often progress insidiously and may remain asymptomatic in the early stages. A painless cervical lymphadenopathy is frequently the first and sometimes the only presenting sign [62]. Other symptoms may include persistent sore throat, dysphagia (difficulty swallowing), otalgia (ear pain), unexplained weight loss, and in laryngeal involvement, hoarseness or vocal changes. Because these tumors preferentially develop in lymphoid-rich areas such as the tonsils and base of the tongue, local symptoms often reflect their anatomical origin [21,63].

Despite their relatively small primary size, HPV-positive tumors frequently present with advanced cervical lymph node metastasis. While the rate of distant metastasis is similar to HPV-negative HNCs, when present they often follow a more aggressive dissemination pattern [64].

By contrast, HPV-negative HNCs usually present with more pronounced local symptoms, including severe throat pain, progressive dysphagia, otalgia, and hoarseness. These cancers, strongly associated with tobacco and alcohol exposure, often involve the oral cavity, larynx, and hypopharynx sites less commonly affected by HPV [65]. They are typically larger, more locally invasive, and diagnosed at later stages, contributing to poorer prognosis compared with their HPV-positive counterparts [66].

### 4.2. Diagnostic Methods and Challenges

Accurate diagnosis of HPV-related HNCs is critical given their distinct prognosis and treatment response [67]. Diagnostic approaches differ from those used in HPV-negative tumors and require a combination of histopathological, molecular, and biomarker-based methods [68].

#### 4.2.1. Key Diagnostic Differences

1. Histopathology and p16 Immunohistochemistry:
  - p16 overexpression is widely used as a surrogate marker of HPV-driven oncogenesis in OPSCC. Although not entirely specific, strong and diffuse nuclear/cytoplasmic p16 staining strongly correlates with high-risk HPV, particularly HPV16 [69,70,71].
  - Morphologically, HPV-positive tumors display a basaloid, non-keratinizing phenotype with less differentiation, whereas HPV-negative tumors more often show keratinizing morphology [72,73].
2. HPV DNA/RNA Testing:
  - DNA-based methods such as polymerase chain reaction (PCR) and in situ hybridization (ISH) detect the presence of high-risk HPV DNA, with PCR offering high sensitivity [74,75].
  - RNA-based methods assess expression of E6/E7 mRNA, confirming transcriptionally active infection and distinguishing transforming infections from latent ones [76,77,78].
3. Comparison with HPV-Negative HNCs:

HPV-negative HNCs rarely show p16 overexpression and typically harbor genomic alterations such as TP53 mutations, especially in smokers. Their absence of HPV biomarkers underscores the need to combine histology with molecular tests for accurate diagnosis [79,80,81].

#### 4.2.2. Emerging Diagnostic Methods for HPV-Related HNCs

As the understanding of HPV-related HNCs evolves, so do the diagnostic techniques used to identify and characterize these cancers. Several emerging methods offer the potential to improve the accuracy and specificity of HPV-related HNC diagnosis [82].

- **Next-Generation Sequencing (NGS):** NGS allows for comprehensive genomic profiling of tumors, providing insights into the genetic landscape of HPV-related HNCs [83]. This technique can identify viral integration sites, mutation signatures, and other molecular features unique to HPV-related tumors. NGS also helps in distinguishing HPV-related from HPV-nonrelated tumors by identifying characteristic mutations [83].
- **Liquid Biopsy:** Analysis of circulating tumor DNA (ctDNA) or circulating HPV DNA (ctHPV DNA) enables non-invasive monitoring, early recurrence detection, and treatment response assessment [84].
- **Circulating Biomarkers:** Beyond ctDNA, circulating biomarkers such as antibodies against HPV oncoproteins (E6 and E7) and miRNAs are being studied for their potential role in the diagnosis and prognosis of HPV-related HNCs. These biomarkers could offer a non-invasive method to monitor disease status and guide therapeutic decisions [85,86,87,88].

#### 4.2.3. Challenges in the Diagnosis of HPV-Related HNCs

- **Asymptomatic Presentation:** Asymptomatic nature of early-stage HPV-related HNCs presents a significant diagnostic challenge. Patients may only present with a painless cervical lymph node, with little to no primary tumor symptoms, complicating early detection efforts [62,63,67].
- **False Positives and Negatives:** While p16 IHC is a valuable tool, it is not infallible. False positives can occur in HPV-nonrelated tumors that exhibit p16 overexpression without the presence of HPV DNA. Conversely, false negatives may arise in HPV-related tumors with low p16 expression. Combining p16 IHC with HPV DNA/RNA testing is essential to mitigate these risks [70].
- **Tumor Heterogeneity:** HPV-related HNCs exhibit considerable heterogeneity, not only in terms of HPV subtypes but also in their biological behavior and response to treatment. This heterogeneity complicates the diagnostic process, necessitating a multifaceted approach that includes histopathology, molecular testing, and biomarker analysis [87,88].

#### 4.2.4. Role of Biomarkers in Diagnosis

Biomarkers play a crucial role in the accurate diagnosis of HPV-related HNCs, offering insights into tumor biology, prognosis, and potential therapeutic targets. As previously discussed, p16 remains the most widely used surrogate marker for HPV in oropharyngeal cancers (OPC) [79]. However, its use should be complemented by other diagnostic tests to confirm HPV status [89,90]. Detection of E6/E7 mRNA provides strong evidence of active HPV infection and oncogenic activity, making it a critical biomarker for confirming HPV-driven carcinogenesis in HNCs [85,86]. To summarize current advances, Table 2 outlines key diagnostic and prognostic biomarkers under investigation in HPV-related HNCs.

Research into additional biomarkers, such as specific miRNAs and proteins, is ongoing. These biomarkers could help stratify patients based on their risk and guide personalized treatment approaches. For example, lower expression of certain miRNAs may be associated with more aggressive disease and poorer outcomes [90,93,94].

The diagnosis of HPV HNCs requires a nuanced approach that integrates clinical evaluation, histopathological assessment, and advanced molecular testing. Emerging diagnostic techniques and biomarkers hold the promise of improving diagnostic accuracy and enabling earlier detection of these cancers. As research continues to unfold, the hope is that these advancements will lead to more personalized and effective treatment strategies for patients with HPV-related HNCs.

## 5. Treatment and Prognosis

The management of HNSCC has evolved significantly with the recognition of distinct subgroups based on HPV status [95]. HPV-positive HNSCCs, particularly those originating in the oropharynx, display unique biological behaviors that influence both treatment strategies and prognosis [96].

### 5.1. Standard Treatment Approaches for HPV-Related HNCs

**Radiotherapy (RT):** RT is often the cornerstone of treatment for HPV-positive OPCs, especially in early-stage disease. These tumors are generally more radiosensitive compared to HPV-negative tumors, which allows for effective tumor control with potentially lower doses. Given their favorable prognosis, treatment de-escalation has been explored to reduce long-term toxicity. Studies have investigated reduced RT doses and omission of concurrent chemotherapy in carefully selected patients [97,98,99].

Two randomized trials showed that replacing cisplatin with cetuximab during definitive radiotherapy leads to inferior outcomes: in RTOG 1016, 5-year OS was 84.6% with cisplatin versus 77.9% with cetuximab (HR for death 1.45), with no overall reduction in moderate-to-severe toxicity; in De-ESCALaTE, 2-year OS was 97.5% vs. 89.4% and 2-year recurrence 6.0% vs. 16.1% favoring cisplatin, again without a toxicity advantage for cetuximab [100,101]. In contrast, NRG-HN002 supported 60 Gy IMRT + weekly cisplatin (2-year PFS 90.5%, 1-year MDADI 85.3), whereas 60 Gy IMRT alone did not meet the PFS benchmark [102]. In the surgery pathway, E3311 (TOS with risk-adapted reduced-dose adjuvant RT) achieved ~95–96% 2-year PFS in intermediate-risk arms with favorable swallowing/QoL outcomes [103].

**Chemoradiotherapy (CRT):** For locally advanced disease, concurrent CRT with cisplatin remains the standard. Platinum-based chemotherapy enhances radiosensitivity, but ongoing research is assessing reduced chemotherapy intensity or alternative agents with fewer side effects [95,104,105,106].

**Surgery:** Minimally invasive techniques such as transoral robotic surgery (TORS) are increasingly used. TORS permits precise resection with minimal morbidity and may be followed by adjuvant RT or CRT depending on pathology [107,108].

### 5.2. Emerging and Investigational Treatment Modalities

Immune checkpoint inhibitors (ICIs) have transformed the management of recurrent/metastatic HNSCC, with pembrolizumab and nivolumab now standard of care. In KEYNOTE-048, pembrolizumab (alone or with platinum/5-FU) improved OS compared with the EXTREME regimen, establishing it as a first-line option [109]. Nivolumab also improved OS in platinum-refractory disease in CheckMate-141 [110], while pembrolizumab showed durable responses in KEYNOTE-012/055 [111,112].

Perioperative immunotherapy is now reshaping curative-intent management. In the phase III KEYNOTE-689 trial, adding neoadjuvant and adjuvant pembrolizumab to standard therapy significantly improved event-free survival in resectable, locally advanced HNSCC an effect most evident in PD-L1 CPS ≥1 disease [113]. On the basis of KEYNOTE-689, the U.S. FDA (12 June 2025) approved perioperative pembrolizumab for appropriate patients. Complementing this, the phase III NIVOPOSTOP (GORTEC 2018-01) study showed that adding adjuvant nivolumab to postoperative radiotherapy in high-risk resected HNSCC improved disease-free survival with a 24% reduction in the risk of recurrence or death. Together, these results position perioperative PD-1 blockade as a pivotal component of curative-intent pathways and support biomarker-guided integration with surgery and radiotherapy.

Novel strategies are being developed on this immunotherapy backbone. Therapeutic vaccines targeting HPV E6/E7 are in clinical testing [114,115,116,117]. CUE-101, an HPV16-specific Immuno-STAT, achieved a 47% ORR in HPV16+ R/M HNSCC [118]. HB-200, an arenavirus-based vector, showed ORRs of 43% with pembrolizumab, rising to 59% in PD-L1 CPS ≥20 patients [119]. Neoadjuvant combinations of HB-200 or sintilimab with chemotherapy have demonstrated high rates of partial and complete responses [120,121]. Dual checkpoint blockade with EGFR inhibitors, such as nivolumab plus cetuximab, has also shown encouraging survival [122]. The ISA101b vaccine combined with cemiplimab improved survival in PD-L1 CPS ≥20 patients [118].

Targeted therapies are being investigated, particularly against PI3K/Akt/mTOR signalling upregulated by E6/E7. While mTOR inhibition alone has shown modest efficacy, ongoing trials are assessing adjuvant benefit [123,124].

De-escalation strategies have been tested, including hypoxia-guided RT dose reduction (30 Gy achieving ~95% 2-year locoregional control) [125], TORS with pathology-driven adjuvant therapy (54-month PFS 90.6%) [126], and intensity-modulated proton therapy (IMPT), which was non-inferior to IMRT with reduced toxicity in a Phase III trial [127].

Liquid biopsy approaches, particularly circulating tumor HPV DNA, are emerging as promising tools for minimal residual disease monitoring. The HPV-DeepSeek assay detected recurrence a median of 207 days before clinical diagnosis and predicted worse PFS in patients with persistent ctHPV DNA [128].

Gene-editing technologies, including CRISPR/Cas9 targeting E6/E7, have induced apoptosis and growth arrest in preclinical models [129,130]. CRISPR/Cas13a has also been shown to degrade E6/E7 transcripts and restore p53/Rb function [131]. Cell-based therapies, including TCR-engineered T cells and CAR-T targeting HPV16 antigens, have shown early efficacy [132,133].

In summary, the treatment paradigm in HPV-related HNSCC is shifting toward personalized, immune- and biomarker-driven approaches. The integration of ICIs, vaccines, molecular targets, and cell therapies is reshaping clinical trial designs, with radiation de-escalation and liquid biopsy technologies further enabling risk-adapted strategies.

### 5.3. Prognosis and Survival Outcomes

HPV-positive HNSCC patients have significantly better prognosis compared with HPV-negative counterparts, largely due to improved radiosensitivity and younger, healthier patient populations [134]. Five-year survival rates for HPV-positive OPSCC often exceed 80%, compared to ~47% in HPV-negative disease [135,136,137].

However, concurrent smoking reduces the survival benefit in HPV-positive patients, leading to outcomes more similar to HPV-negative disease. This highlights the importance of integrating smoking cessation into treatment plans [136]. HPV-negative HNSCCs, usually associated with tobacco and alcohol, present later, are more aggressive, and often require more intensive treatment, resulting in higher toxicity and worse quality of life [95,137].

## 6. HPV Vaccines and Prevention of HPV-Related HNCs

Vaccination against HPV represents one of the most significant public health measures for preventing HPV-associated diseases, including HNCs [138]. Initially, global vaccination programs focused on cervical cancer, which is directly linked to persistent HPV infection [139,140]. However, as evidence has accumulated confirming HPV’s etiological role in OPCs, the preventive potential of vaccines has expanded to encompass HNCs [141,142]. Current vaccines demonstrate high efficacy against the most common high-risk HPV types, particularly HPV16 and HPV18, which are responsible for the majority of HPV-related malignancies [143].

To date, three prophylactic vaccines have been approved. Gardasil (quadrivalent) targets HPV types 6, 11, 16, and 18, while Cervarix (bivalent) protects against HPV16 and HPV18. The most recent, Gardasil9 (nonavalent), covers HPV types 6, 11, 16, 18, 31, 33, 45, 52, and 58, providing broader protection [144,145]. In the United States, the 9-valent vaccine is the standard and is recommended for prevention of multiple HPV-associated cancers, including HNCs [139]. These vaccines are prophylactic rather than therapeutic, designed to prevent infection before viral exposure. They work by priming humoral immunity using virus-like particles (VLPs) derived from the L1 capsid protein, which elicit antibody levels up to 100-fold higher than those induced by natural infection [45,146].

### 6.1. Therapeutic Vaccines

While prophylactic vaccines have successfully reduced infection rates and precancerous lesions, they are ineffective against established HPV infections or HPV-driven neoplasms. Consequently, therapeutic vaccine development remains an important area of research [146]. Current efforts focus on targeting the viral oncoproteins E6 and E7, which are essential for malignant transformation. However, despite promising preclinical data, clinical trials have struggled to elicit durable immune responses [147,148].

Alternative strategies are exploring vaccines against other viral proteins, such as L2, as well as innovative platforms including DNA- and vector-based vaccines. Combining therapeutic vaccines with immune checkpoint inhibitors has also demonstrated encouraging results, suggesting synergistic effects that may enhance tumor-specific immunity [120,149,150].

### 6.2. Effectiveness in Preventing HNCs

Although HPV vaccines were developed primarily for cervical and anogenital cancers, mounting evidence supports their role in preventing HNCs, particularly OPCs, where HPV16 is the predominant driver [12,150,151]. Several studies demonstrate reduced oral HPV infections following vaccination, a critical step in preventing OPC. For instance, a meta-analysis showed vaccinated individuals were 46% less likely to acquire oral HPV, with vaccine efficacy against HPV16 and HPV18 infections reaching up to 93.3% [151]. Regulatory agencies, including the WHO and U.S. FDA, now acknowledge the role of HPV vaccines in reducing the incidence of HPV-related HNCs [151]. Importantly, the inclusion of HNSCC among the conditions preventable by the 9-valent vaccine further highlights its clinical impact [152,153].

### 6.3. Vaccination Strategies and Global Challenges

The most effective HPV vaccination strategies target children aged 9–14, before sexual debut, ensuring immunity prior to viral exposure [154]. Nevertheless, implementation varies widely worldwide, with low- and middle-income countries facing major obstacles such as limited healthcare infrastructure, vaccine costs, and misinformation [155,156,157]. For example, in Kenya, only one-third of eligible girls received the first vaccine dose at program initiation in 2019, and fewer than 20% completed the two-dose series [156]. In China, coverage remains uneven, while in Turkey, the vaccine is not yet included in the national immunization schedule, resulting in low uptake and awareness [158,159].

Vaccine hesitancy also plays a significant role, often driven by cultural barriers, stigma around sexual activity, or concerns about side effects [157,160]. Studies in Latin America and Turkey reveal low awareness and misconceptions about HPV and its vaccines, with fears about safety contributing to poor acceptance [158,161,162]. Addressing these issues requires educational campaigns, engagement with healthcare professionals, and targeted community outreach [163,164]. Equally important is emphasizing the need to vaccinate boys as well as girls, since HPV affects both sexes.

Although HPV vaccination was initially restricted to adolescents, programs have expanded to include adults. In the United States, the FDA has approved vaccination for individuals aged 27–45, particularly those not previously exposed to the virus [160,163]. While catch-up vaccination can provide benefits, its effectiveness may be reduced due to prior HPV exposure and immune system decline with age, and accessibility remains limited by financial and logistical barriers [155].

## 7. Ongoing Research into HNCs—Related to HPV

HPV is a well-established etiological driver of HNCs; with its role most clearly demonstrated in OPSCC. Nevertheless, research efforts are increasingly extending beyond OPSCC to evaluate the contribution of HPV to other subsites, including the larynx, hypopharynx, sinonasal cavity, nasopharynx, and salivary glands. These investigations seek to clarify the molecular mechanisms underlying HPV-driven carcinogenesis in these regions, to define the clinical and prognostic implications of viral involvement, and to identify opportunities for therapeutic innovation in cancer types where the oncogenic role of HPV remains less well characterized.

### 7.1. Oropharyngeal Cancer

High-risk HPV is a major cause of OPSCC, which has now surpassed cervical cancer in the United States to become the most common HPV-related malignancy [144,165]. Among HPV-positive OPSCC, HPV16 accounts for >80–90% of cases, whereas HPV18 is detected in ~2–3% and HPV33 is slightly more frequent than HPV18 [166]. In North America, HPV16 positivity has been reported in 40–80% of OPSCC cases, while prevalence rates in non-oropharyngeal HNCs remain lower (5–20%), with some regional variability [167].

Epidemiological studies indicate that the rising incidence of HPV-positive OPSCC in Western countries is strongly linked to changing sexual behaviors, particularly the increasing prevalence of oral sexual practices over the past five decades [168,169]. While HPV plays a well-established etiological role in OPSCC, its oncogenic contribution in oral cavity cancers remains less clear and continues to be debated [170].

Importantly, transcriptionally active HPV infection, demonstrated by the presence of E6/E7 mRNA, is critical for accurate diagnosis and clinical management, as HPV-positive OPSCC is associated with better prognosis and distinct therapeutic implications [171]. Kaplan–Meier analyses from large cohorts consistently show that patients with HPV-positive tumors have significantly better overall survival compared with HPV-negative counterparts [172]. This favorable prognosis has stimulated the design of treatment de-intensification strategies, which aim to reduce long-term toxicities without compromising cure rates. For example, clinical studies support the feasibility of lowering radiation or chemotherapy intensity in selected HPV-positive OPSCC patients, while maintaining the therapeutic contribution of cisplatin for local control [173].

Moreover, the strong viral antigenicity of HPV-positive tumors has provided a rationale for integrating immunotherapy into earlier stages of treatment. Recent trials of neoadjuvant immune checkpoint inhibitors in OPSCC suggest that these agents may permit reduced doses of surgery, radiotherapy, or chemotherapy, thereby improving quality of life while preserving survival outcomes [174].

### 7.2. Laryngeal Cancer

The role of HPV in laryngeal squamous cell carcinoma (LSCC) is less clearly established than in OPSCC, yet several studies suggest potential clinical relevance. HPV-positive and PD-L1–positive LSCC patients demonstrated better overall survival, with PD-L1 positivity more frequent in non-smokers. These findings indicate that HPV and PD-L1 status may represent independent favorable prognostic factors and support exploration of immune checkpoint inhibitors in LSCC [174].

By contrast, other investigations highlight the rarity of HPV involvement. In one analysis of 78 LSCC samples, HPV DNA was detected in only 9%, and p16 was not considered a reliable surrogate marker [89]. However, retrospective studies have shown that p16-positive LSCC is associated with improved survival, suggesting a possible distinct subgroup [175].

A systematic review of 77 studies spanning nearly a century emphasized the overall low prevalence of HPV in LSCC but highlighted the need for advanced models to rigorously study high-risk HPV types [176].

### 7.3. Hypopharyngeal Cancer

Emerging evidence suggests that HPV positivity may confer a survival advantage in hypopharyngeal squamous cell carcinoma (HPSCC). In one cohort of 108 patients, HPV16-positive tumors exhibited significantly higher tumor-infiltrating lymphocyte (TIL) density and improved three-year survival rates compared with HPV-negative tumors [177].

Large-scale analyses support this trend. Data from the U.S. National Cancer Database (NCDB) including more than 9000 patients showed that HPV-positive HPSCC was associated with improved survival regardless of treatment modality [178]. Another NCDB study (2010–2017) confirmed the independent prognostic value of HPV status, underscoring its importance in this aggressive cancer subtype [179].

### 7.4. Sinonasal Cancer

HPV contributes to a subset of sinonasal squamous cell carcinomas (SNSCC). Recent studies estimate that 25–30% of SNSCC cases are HPV-positive, with HPV16 the predominant genotype [180]. A systematic review of 57 studies reported that HPV-positive SNSCC consistently correlated with better overall and progression-free survival [181].

Histological analyses show broad morphological diversity. In a series of 153 tumors, 18% of HPV-related SNSCCs were classified as non-keratinizing or partially keratinizing SCCs, underscoring heterogeneity [182]. Nonetheless, the reliability of p16 as a surrogate marker remains debated, with some studies finding discordance between p16 and HPV DNA/RNA [183]. Overall, HPV positivity in SNSCC appears to confer a prognostic advantage, but standardization of diagnostic criteria is needed.

### 7.5. Nasopharyngeal Cancer

Although Epstein–Barr virus (EBV) is the main viral driver in nasopharyngeal carcinoma (NPC), HPV has been implicated in a subset of cases. A systematic review and meta-analysis of 46 studies (6314 patients) reported that HPV-positive NPC has a global prevalence of ~18%, with the highest rates in North America (25%) and the lowest in Asia (13%). HPV16 and HPV18 were the most frequent genotypes, and most HPV-positive NPCs were classified as WHO Type I keratinizing squamous cell carcinoma [180].

Circulating biomarkers are emerging in this setting. ctHPV DNA has shown 100% sensitivity at diagnosis and detected recurrence earlier than clinical evaluation, highlighting its utility for disease monitoring [180]. Population-based studies also suggest that prior HPV infection significantly increases the risk of NPC, supporting HPV as a potential co-factor with EBV [89]. Case reports further demonstrate that ctHPV DNA levels track with tumor burden and relapse, offering promise for personalized follow-up [127].

### 7.6. Salivary Gland Cancer

The role of HPV in salivary gland cancers remains uncertain. A population-based analysis of 416 patients and 2080 controls found that HPV infection was significantly more common in salivary gland cancer patients [181]. However, other studies have failed to confirm this association. For instance, a Sudanese study reported high HPV16 prevalence in esophageal and laryngeal cancers but low prevalence in salivary gland tumors, suggesting that HPV16 is unlikely to be a consistent driver [182].

A systematic review concluded that while HPV may play a role in specific subtypes such as mucoepidermoid carcinoma, its involvement across salivary gland malignancies is not definitive [183]. Larger molecularly characterized cohorts are needed to resolve whether HPV detection in these tumors represents a causal role or incidental infection.

In conclusion, the impact of HPV on HNCs extends well beyond OPSCC, with varying degrees of involvement across laryngeal, hypopharyngeal, sinonasal, nasopharyngeal, and salivary gland cancers. While its etiological role is firmly established in OPSCC, the oncogenic significance of HPV in other subsites remains less clear and requires further research. Notably, in some subtypes such as hypopharyngeal squamous cell carcinoma and sinonasal carcinoma, HPV positivity appears to confer a survival advantage, potentially linked to enhanced immune responses. In contrast, its contribution to salivary gland cancers remains controversial. Defining the molecular mechanisms and prognostic implications of HPV across these tumor types will be essential for refining diagnostic accuracy, developing novel biomarkers, and tailoring treatment strategies. Future investigations should prioritize understanding site-specific tumor biology and integrating viral, epigenetic, and immune features into optimized therapeutic approaches.

## 8. Conclusions and Future Directions

HPV is now firmly established as a critical etiological factor in HNCs, particularly OPSCC. With the rising global incidence of HPV-related HNCs, deeper understanding of its role in tumorigenesis remains essential to guide both research and clinical practice. This review has highlighted the complex interactions between viral oncoproteins, host pathways, and epigenetic mechanisms that drive malignant transformation.

HPV-positive HNCs display distinct epidemiological, clinical, and molecular profiles compared with HPV-negative disease, underscoring the need for tailored diagnostic and therapeutic strategies. Current diagnostic tools rely heavily on p16 immunohistochemistry and HPV DNA/RNA assays, while emerging approaches such as liquid biopsy and next-generation sequencing promise earlier detection and better disease monitoring. However, challenges remain, including tumor heterogeneity, asymptomatic presentation, and limitations of surrogate markers. Expanding the use of circulating biomarkers (e.g., ctHPV DNA, microRNAs) may improve diagnostic precision and risk stratification.

Treatment strategies for HPV-positive HNCs are shifting toward de-intensification, reflecting their favorable prognosis and enhanced radiosensitivity. Clinical trial data show that reduced-dose radiotherapy or risk-adapted adjuvant therapy can maintain excellent survival while lowering toxicity. Concurrently, immunotherapy, therapeutic vaccines targeting E6/E7, and targeted molecular agents are broadening the treatment landscape, offering opportunities to combine efficacy with long-term quality-of-life preservation.

HPV vaccination is one of the most effective preventive measures against HPV-driven cancers. While high-income countries have achieved notable success, global uptake remains uneven due to cost, infrastructure, and hesitancy. Broader implementation including programs targeting both girls and boys, catch-up vaccination for adults, and culturally tailored education will be essential to reduce the future burden of HPV-related HNCs.

Looking ahead, several key research priorities are emerging in the field of HPV-related HNCs. First, there is a need to expand the focus beyond OPSCC to better understand the role of HPV in less common subtypes, including laryngeal, hypopharyngeal, sinonasal, nasopharyngeal, and salivary gland cancers, where its contribution remains poorly defined. Another important direction involves clarifying the prognostic and therapeutic implications of viral genome status specifically the distinction between episomal and integrated HPV genomes which could serve as a valuable biomarker for patient stratification and treatment planning. In addition, demographic shifts are becoming increasingly evident, with trends in age distribution, likely linked to birth cohort effects, as well as sex-based differences in incidence and clinical outcomes, both of which warrant systematic investigation. Finally, the integration of molecular biomarkers such as epigenetic alterations, non-coding RNAs, and immune signatures into clinical practice holds great promise for refining risk assessment and advancing personalized therapeutic approaches.

In conclusion, HPV-related HNCs represent both a challenge and an opportunity. Continued progress in prevention, early detection, and personalized treatment will depend on multidisciplinary collaboration between researchers, clinicians, and policymakers. If vaccination programs are expanded and novel therapeutic strategies continue to mature, substantial reductions in HPV-related cancer incidence and improved outcomes for patients worldwide are achievable.

## Figures and Tables

**Table 2:** Emerging Diagnostic and Prognostic Biomarkers in HPV-related HNCs.

Biomarker Type	Example(s)	Clinical/Research Relevance	References
Immunohistochemical markers	p16 overexpression	Widely used surrogate marker for HPV status in OPSCC; correlates with prognosis, but not fully specific	[69,71]
Viral DNA/RNA detection	HPV16/18 DNA (PCR, ISH), E6/E7 mRNA	Confirms transcriptionally active HPV infection; higher specificity than p16 alone	[74,75,76,77,78]
Circulating biomarkers	Anti-E6/E7 antibodies, circulating HPV DNA (ctHPV DNA)	Useful for early detection, monitoring recurrence, and minimal residual disease assessment	[84,85]
Epigenetic alterations	DNA methylation (e.g., L1, E2BS), histone modification	Correlates with disease stage, aggressiveness, and survival; potential for stratification and prognosis	[47,50,52,60,61]
Non-coding RNAs	lncRNAs (CCEPR, FAM83H-AS1, PRINS), miRNAs	Influence proliferation, apoptosis, immune response; emerging as diagnostic/prognostic tools	[54,55,59,91,92]

## Abbreviations

The following abbreviations are used in this manuscript:

HNCs	Head and neck cancers
9vHPV	9-valent HPV vaccine
CDK	Cyclin-dependent kinase
circRNAs	Circular RNAs
CRT	Chemoradiotherapy
CTCF	CCCTC-binding factor
ctDNA	Circulating tumor DNA
E2BS	E2 binding sites
FDA	Food and Drug Administration
HNSCCs	Head and neck squamous cell carcinomas
HPSCC	Hypopharyngeal squamous cell carcinoma
HPV	Human papillomavirus
HPV+NPC	HPV-related nasopharyngeal carcinoma
ICIs	Immune checkpoint inhibitors
IMPT	Intensity-modulated proton therapy
ISH	In situ hybridization
LCR	The long control region
lncRNAs	Long non-coding RNAs
LSCC	Laryngeal squamous cell carcinoma
me	Methylation
miRNAs	MicroRNAs
MRD	Minimal residual disease
NCDB	National Cancer Database
ncRNAs	Non-coding RNAs
NGS	Next-generation sequencing
OPC	Oropharyngeal cancers
OPSCC	Oropharyngeal squamous cell carcinoma
OSCC	Oral squamous cell carcinoma
PCR	Polymerase chain reaction
PFS	Progression-free survival
pRb	Retinoblastoma protein
R/M HNSCC	Recurrent/metastatic head and neck squamous cell carcinoma
RT	Radiotherapy
SETD2	SET-domain containing protein 2
SIRT1	Sirtuin1
SNSCC	Sinonasal squamous cell carcinoma
TORS	Transoral Robotic Surgery
URR	Upstream regulatory region
VLPs	Virus-like particles
WHO	World Health Organization
WRN	Werner Syndrome Protein
YY1	Yin Yang 1

## References

[1] Bray F., Laversanne M., Sung H., Ferlay J., Siegel R.L., Soerjomataram I., Jemal A. (2024). Global Cancer Statistics 2022: GLOBOCAN Estimates of Incidence and Mortality Worldwide for 36 Cancers in 185 Countries. CA Cancer J. Clin..

[2] Mody M.D., Rocco J.W., Yom S.S., Haddad R.I., Saba N.F. (2021). Head and Neck Cancer. Lancet.

[3] Gormley M., Creaney G., Schache A., Ingarfield K., Conway D.I. (2022). Reviewing the Epidemiology of Head and Neck Cancer: Definitions, Trends and Risk Factors. Br. Dent. J..

[4] Anderson G., Ebadi M., Vo K., Novak J., Govindarajan A., Amini A. (2021). An Updated Review on Head and Neck Cancer Treatment with Radiation Therapy. Cancers.

[5] Menezes F.d.S., Fernandes G.A., Antunes J.L.F., Villa L.L., Toporcov T.N. (2021). Global Incidence Trends in Head and Neck Cancer for HPV-Related and -Unrelated Subsites: A Systematic Review of Population-Based Studies. Oral Oncol..

[6] Pytynia K.B., Dahlstrom K.R., Sturgis E.M. (2014). Epidemiology of HPV-Associated Oropharyngeal Cancer. Oral Oncol..

[7] Ndiaye C., Mena M., Alemany L., Arbyn M., Castellsagué X., Laporte L., Bosch F.X., de Sanjosé S., Trottier H. (2014). HPV DNA, E6/E7 MRNA, and P16INK4a Detection in Head and Neck Cancers: A Systematic Review and Meta-Analysis. Lancet Oncol..

[8] Hoppe-Seyler K., Bossler F., Braun J.A., Herrmann A.L., Hoppe-Seyler F. (2018). The HPV E6/E7 Oncogenes: Key Factors for Viral Carcinogenesis and Therapeutic Targets. Trends Microbiol..

[9] Moody C.A., Laimins L.A. (2010). Human Papillomavirus Oncoproteins: Pathways to Transformation. Nat. Rev. Cancer.

[10] Doorbar J., Quint W., Banks L., Bravo I.G., Stoler M., Broker T.R., Stanley M.A. (2012). The Biology and Life-Cycle of Human Papillomaviruses. Vaccine.

[11] Yu V.X., Long S., Tassler A. (2023). Smoking and Head and Neck Cancer. JAMA Otolaryngol.-Head Neck Surg..

[12] Ndon S., Singh A., Ha P.K., Aswani J., Chan J.Y.-K., Xu M.J. (2023). Human Papillomavirus-Associated Oropharyngeal Cancer: Global Epidemiology and Public Policy Implications. Cancers.

[13] Chaturvedi A.K., Engels E.A., Pfeiffer R.M., Hernandez B.Y., Xiao W., Kim E., Jiang B., Goodman M.T., Sibug-Saber M., Cozen W. (2011). Human Papillomavirus and Rising Oropharyngeal Cancer Incidence in the United States. J. Clin. Oncol..

[14] Mehanna H., Taberna M., von Buchwald C., Tous S., Brooks J., Mena M., Morey F., Grønhøj C., Rasmussen J.H., Garset-Zamani M. (2023). Prognostic Implications of P16 and HPV Discordance in Oropharyngeal Cancer (HNCIG-EPIC-OPC): A Multicentre, Multinational, Individual Patient Data Analysis. Lancet Oncol..

[15] Syrjänen S. (2010). The Role of Human Papillomavirus Infection in Head and Neck Cancers. Ann. Oncol..

[16] Vani N.V., Madhanagopal R., Swaminathan R., Ganesan T.S. (2023). Dynamics of Oral Human Papillomavirus Infection in Healthy Population and Head and Neck Cancer. Cancer Med..

[17] Anna Szymonowicz K., Chen J. (2020). Biological and Clinical Aspects of HPV-Related Cancers. Cancer Biol. Med..

[18] Powell S.F., Vu L., Spanos W.C., Pyeon D. (2021). The Key Differences between Human Papillomavirus-Positive and -Negative Head and Neck Cancers: Biological and Clinical Implications. Cancers.

[19] Kobayashi K., Hisamatsu K., Suzui N., Hara A., Tomita H., Miyazaki T. (2018). A Review of HPV-Related Head and Neck Cancer. J. Clin. Med..

[20] Thompson L.D.R. (2020). HPV-Related Multiphenotypic Sinonasal Carcinoma. Ear Nose Throat J..

[21] Lim Y.X., Mierzwa M.L., Sartor M.A., D’Silva N.J. (2023). Clinical, Morphologic and Molecular Heterogeneity of HPV-Associated Oropharyngeal Cancer. Oncogene.

[22] Burley M., Roberts S., Parish J.L. (2020). Epigenetic Regulation of Human Papillomavirus Transcription in the Productive Virus Life Cycle. Semin. Immunopathol..

[23] Bernard H.-U., Burk R.D., Chen Z., van Doorslaer K., Hausen H.z., de Villiers E.-M. (2010). Classification of Papillomaviruses (PVs) Based on 189 PV Types and Proposal of Taxonomic Amendments. Virology.

[24] Bernard H., Calleja-Macias I.E., Dunn S.T. (2006). Genome Variation of Human Papillomavirus Types: Phylogenetic and Medical Implications. Int. J. Cancer.

[25] McBride A.A. (2013). The Papillomavirus E2 Proteins. Virology.

[26] Han F., Guo X., Jiang M., Xia N., Gu Y., Li S. (2024). Structural Biology of the Human Papillomavirus. Structure.

[27] Ullah M.I., Mikhailova M.V., Alkhathami A.G., Carbajal N.C., Zuta M.E.C., Rasulova I., Najm M.A.A., Abosoda M., Alsalamy A., Deorari M. (2023). Molecular Pathways in the Development of HPV-Induced Oropharyngeal Cancer. Cell Commun. Signal..

[28] Ashrafi G.H., Salman N.A. (2016). Pathogenesis of Human Papillomavirus—Immunological Responses to HPV Infection. Human Papillomavirus—Research in a Global Perspective.

[29] Kombe Kombe A.J., Li B., Zahid A., Mengist H.M., Bounda G.-A., Zhou Y., Jin T. (2021). Epidemiology and Burden of Human Papillomavirus and Related Diseases, Molecular Pathogenesis, and Vaccine Evaluation. Front. Public. Health.

[30] McBride A.A., Warburton A. (2017). The Role of Integration in Oncogenic Progression of HPV-Associated Cancers. PLoS Pathog..

[31] Parfenov M., Pedamallu C.S., Gehlenborg N., Freeman S.S., Danilova L., Bristow C.A., Lee S., Hadjipanayis A.G., Ivanova E.V., Wilkerson M.D. (2014). Characterization of HPV and Host Genome Interactions in Primary Head and Neck Cancers. Proc. Natl. Acad. Sci. USA.

[32] Groves I.J., Tang G., Pentland I., Parish J.L., Coleman N. (2021). CTCF Association with Episomal HPV16 Genomes Regulates Viral Oncogene Transcription and Splicing. bioRxiv.

[33] Mac M., Moody C.A. (2020). Epigenetic Regulation of the Human Papillomavirus Life Cycle. Pathogens.

[34] Mac M., DeVico B.M., Raspanti S.M., Moody C.A. (2023). The SETD2 Methyltransferase Supports Productive HPV31 Replication through the LEDGF/CtIP/Rad51 Pathway. J. Virol..

[35] Ashrafi G.H., Tsirimonaki E., Marchetti B., O’Brien P.M., Sibbet G.J., Andrew L., Campo M.S. (2002). Down-Regulation of MHC Class I by Bovine Papillomavirus E5 Oncoproteins. Oncogene.

[36] Faraji F., Zaidi M., Fakhry C., Gaykalova D.A. (2017). Molecular Mechanisms of Human Papillomavirus-Related Carcinogenesis in Head and Neck Cancer. Microbes Infect..

[37] Vats A., Trejo-Cerro O., Thomas M., Banks L. (2021). Human Papillomavirus E6 and E7: What Remains?. Tumour Virus Res..

[38] Lim J., Lilie H., Kalbacher H., Roos N., Frecot D.I., Feige M., Conrady M., Votteler T., Cousido-Siah A., Corradini Bartoli G. (2023). Evidence for Direct Interaction between the Oncogenic Proteins E6 and E7 of High-Risk Human Papillomavirus (HPV). J. Biol. Chem..

[39] Klingelhutz A.J., Roman A. (2012). Cellular Transformation by Human Papillomaviruses: Lessons Learned by Comparing High- and Low-Risk Viruses. Virology.

[40] Liu X., Dakic A., Zhang Y., Dai Y., Chen R., Schlegel R. (2009). HPV E6 Protein Interacts Physically and Functionally with the Cellular Telomerase Complex. Proc. Natl. Acad. Sci. USA.

[41] Boyer S.N., Wazer D.E., Band V. (1996). E7 Protein of Human Papilloma Virus-16 Induces Degradation of Retinoblastoma Protein through the Ubiquitin-Proteasome Pathway. Cancer Res..

[42] Dyson N., Howley P.M., Münger K., Harlow E. (1989). The Human Papilloma Virus-16 E7 Oncoprotein Is Able to Bind to the Retinoblastoma Gene Product. Science.

[43] Skelin J., Sabol I., Tomaić V. (2022). Do or Die: HPV E5, E6 and E7 in Cell Death Evasion. Pathogens.

[44] Cosper P.F., Bradley S., Luo Q., Kimple R.J. (2021). Biology of HPV Mediated Carcinogenesis and Tumor Progression. Semin. Radiat. Oncol..

[45] Mo Y., Ma J., Zhang H., Shen J., Chen J., Hong J., Xu Y., Qian C. (2022). Prophylactic and Therapeutic HPV Vaccines: Current Scenario and Perspectives. Front. Cell. Infect. Microbiol..

[46] McBride A.A. (2022). Human Papillomaviruses: Diversity, Infection and Host Interactions. Nat. Rev. Microbiol..

[47] Castro-Oropeza R., Piña-Sánchez P. (2022). Epigenetic and Transcriptomic Regulation Landscape in HPV+ Cancers: Biological and Clinical Implications. Front. Genet..

[48] Pentland I., Campos-León K., Cotic M., Davies K.-J., Wood C.D., Groves I.J., Burley M., Coleman N., Stockton J.D., Noyvert B. (2018). Disruption of CTCF-YY1–Dependent Looping of the Human Papillomavirus Genome Activates Differentiation-Induced Viral Oncogene Transcription. PLoS Biol..

[49] James C.D., Das D., Morgan E.L., Otoa R., Macdonald A., Morgan I.M. (2020). Werner Syndrome Protein (WRN) Regulates Cell Proliferation and the Human Papillomavirus 16 Life Cycle during Epithelial Differentiation. mSphere.

[50] Nakagawa T., Kurokawa T., Mima M., Imamoto S., Mizokami H., Kondo S., Okamoto Y., Misawa K., Hanazawa T., Kaneda A. (2021). DNA Methylation and HPV-Associated Head and Neck Cancer. Microorganisms.

[51] von Knebel Doeberitz M., Prigge E.-S. (2019). Role of DNA Methylation in HPV Associated Lesions. Papillomavirus Res..

[52] Clarke M.A., Wentzensen N., Mirabello L., Ghosh A., Wacholder S., Harari A., Lorincz A., Schiffman M., Burk R.D. (2012). Human Papillomavirus DNA Methylation as a Potential Biomarker for Cervical Cancer. Cancer Epidemiol. Biomark. Prev..

[53] Yenigul N.N., Yazıcı Yılmaz F., Ayhan I. (2021). Can Serum Vitamin B12 and Folate Levels Predict HPV Penetration in Patients with ASCUS?. Nutr. Cancer.

[54] Casarotto M., Fanetti G., Guerrieri R., Palazzari E., Lupato V., Steffan A., Polesel J., Boscolo-Rizzo P., Fratta E. (2020). Beyond MicroRNAs: Emerging Role of Other Non-Coding RNAs in HPV-Driven Cancers. Cancers.

[55] Bonelli P., Borrelli A., Tuccillo F.M., Buonaguro F.M., Tornesello M.L. (2021). The Role of CircRNAs in Human Papillomavirus (HPV)-Associated Cancers. Cancers.

[56] Sharma S., Munger K. (2018). Expression of the Cervical Carcinoma Expressed PCNA Regulatory (CCEPR) Long Noncoding RNA Is Driven by the Human Papillomavirus E6 Protein and Modulates Cell Proliferation Independent of PCNA. Virology.

[57] Barr J.A., Hayes K.E., Brownmiller T., Harold A.D., Jagannathan R., Lockman P.R., Khan S., Martinez I. (2019). Long Non-Coding RNA FAM83H-AS1 Is Regulated by Human Papillomavirus 16 E6 Independently of P53 in Cervical Cancer Cells. Sci. Rep..

[58] Kopczyńska M., Kolenda T., Guglas K., Sobocińska J., Teresiak A., Bliźniak R., Mackiewicz A., Mackiewicz J., Lamperska K. (2020). PRINS LncRNA Is a New Biomarker Candidate for HPV Infection and Prognosis of Head and Neck Squamous Cell Carcinomas. Diagnostics.

[59] Dias T.R., Santos J.M.O., Gil da Costa R.M., Medeiros R. (2021). Long Non-Coding RNAs Regulate the Hallmarks of Cancer in HPV-Induced Malignancies. Crit. Rev. Oncol. Hematol..

[60] Hinić S., Rich A., Anayannis N.V., Cabarcas-Petroski S., Schramm L., Meneses P.I. (2022). Gene Expression and DNA Methylation in Human Papillomavirus Positive and Negative Head and Neck Squamous Cell Carcinomas. Int. J. Mol. Sci..

[61] Luo H., Lian Y., Tao H., Zhao Y., Wang Z., Zhou J., Zhang Z., Jiang S. (2024). Relationship between P16/Ki67 Immunoscores and PAX1/ZNF582 Methylation Status in Precancerous and Cancerous Cervical Lesions in High-Risk HPV-Positive Women. BMC Cancer.

[62] Batool S., Sethi R.K.V., Wang A., Dabekaussen K., Egloff A.M., Del Vecchio Fitz C., Kuperwasser C., Uppaluri R., Shin J., Rettig E.M. (2023). Circulating Tumor-Tissue Modified HPV DNA Testing in the Clinical Evaluation of Patients at Risk for HPV-Positive Oropharynx Cancer: The IDEA-HPV Study. Oral Oncol..

[63] Fahmy M.D., Clegg D., Belcastro A., Smith B.D., Eric Heidel R., Carlson E.R., Hechler B. (2022). Are Throat Pain and Otalgia Predictive of Perineural Invasion in Squamous Cell Carcinoma of the Oropharynx?. J. Oral Maxillofac. Surg..

[64] Rajasekaran K., Carey R.M., Lin X., Seckar T.D., Wei Z., Chorath K., Newman J.G., O’Malley B.W., Weinstein G.S., Feldman M.D. (2021). The Microbiome of HPV-Positive Tonsil Squamous Cell Carcinoma and Neck Metastasis. Oral Oncol..

[65] Yuan Y., Huang J., Cao J., Wu J., Wang L., Gan H., Xu J., Ye F. (2025). Tobacco and Alcohol Use Are the Risk Factors Responsible for the Greatest Burden of Head and Neck Cancers: A Study from the Global Burden of Disease Study 2019. Ann. Med..

[66] McIlwain W.R., Sood A.J., Nguyen S.A., Day T.A. (2014). Initial Symptoms in Patients With HPV-Positive and HPV-Negative Oropharyngeal Cancer. JAMA Otolaryngol.-Head Neck Surg..

[67] Blitzer G.C., Smith M.A., Harris S.L., Kimple R.J. (2014). Review of the Clinical and Biologic Aspects of Human Papillomavirus-Positive Squamous Cell Carcinomas of the Head and Neck. Int. J. Radiat. Oncol. Biol. Phys..

[68] Amaral M.N., Faísca P., Ferreira H.A., Gaspar M.M., Reis C.P. (2022). Current Insights and Progress in the Clinical Management of Head and Neck Cancer. Cancers.

[69] Gillison M.L. (2000). Evidence for a Causal Association Between Human Papillomavirus and a Subset of Head and Neck Cancers. J. Natl. Cancer Inst..

[70] Simoens C., Gheit T., Ridder R., Gorbaslieva I., Holzinger D., Lucas E., Rehm S., Vermeulen P., Lammens M., Vanderveken O.M. (2022). Accuracy of High-Risk HPV DNA PCR, P16(INK4a) Immunohistochemistry or the Combination of Both to Diagnose HPV-Driven Oropharyngeal Cancer. BMC Infect. Dis..

[71] Gallus R., Nauta I.H., Marklund L., Rizzo D., Crescio C., Mureddu L., Tropiano P., Delogu G., Bussu F. (2023). Accuracy of P16 IHC in Classifying HPV-Driven OPSCC in Different Populations. Cancers.

[72] Cubilla A.L., Lloveras B., Alejo M., Clavero O., Chaux A., Kasamatsu E., Velazquez E.F., Lezcano C., Monfulleda N., Tous S. (2010). The Basaloid Cell Is the Best Tissue Marker for Human Papillomavirus in Invasive Penile Squamous Cell Carcinoma: A Study of 202 Cases From Paraguay. Am. J. Surg. Pathol..

[73] Oppel F., Gendreizig S., Martinez-Ruiz L., Florido J., López-Rodríguez A., Pabla H., Loganathan L., Hose L., Kühnel P., Schmidt P. (2024). Mucosa-like Differentiation of Head and Neck Cancer Cells Is Inducible and Drives the Epigenetic Loss of Cell Malignancy. Cell Death Dis..

[74] Chaux A., Sanchez D.F., Fernández-Nestosa M.J., Cañete-Portillo S., Rodríguez I.M., Giannico G.A., Cubilla A.L. (2022). The Dual Pathogenesis of Penile Neoplasia: The Heterogeneous Morphology of Human Papillomavirus-Related Tumors. Asian J. Urol..

[75] Baněčková M., Cox D. (2023). Top 10 Basaloid Neoplasms of the Sinonasal Tract. Head Neck Pathol..

[76] Giorgi Rossi P., Ronco G., Mancuso P., Carozzi F., Allia E., Bisanzi S., Gillio-Tos A., De Marco L., Rizzolo R., Gustinucci D. (2022). Performance of HPV E6/E7 MRNA Assay as Primary Screening Test: Results from the NTCC2 Trial. Int. J. Cancer.

[77] Suresh K., Shah P.V., Coates S., Alexiev B.A., Samant S. (2021). In Situ Hybridization for High Risk HPV E6/E7 MRNA in Oropharyngeal Squamous Cell Carcinoma. Am. J. Otolaryngol..

[78] Giorgi Rossi P., Carozzi F., Ronco G., Allia E., Bisanzi S., Gillio-Tos A., De Marco L., Rizzolo R., Gustinucci D., Del Mistro A. (2021). P16/Ki67 and E6/E7 MRNA Accuracy and Prognostic Value in Triaging HPV DNA-Positive Women. JNCI J. Natl. Cancer Inst..

[79] Klussmann J.P., Gültekin E., Weissenborn S.J., Wieland U., Dries V., Dienes H.P., Eckel H.E., Pfister H.J., Fuchs P.G. (2003). Expression of P16 Protein Identifies a Distinct Entity of Tonsillar Carcinomas Associated with Human Papillomavirus. Am. J. Pathol..

[80] Blons H., Laurent-Puig P. (2003). TP53 and Head and Neck Neoplasms. Hum. Mutat..

[81] Qin T., Li S., Henry L.E., Liu S., Sartor M.A. (2021). Molecular Tumor Subtypes of HPV-Positive Head and Neck Cancers: Biological Characteristics and Implications for Clinical Outcomes. Cancers.

[82] Krsek A., Baticic L., Braut T., Sotosek V. (2024). The Next Chapter in Cancer Diagnostics: Advances in HPV-Positive Head and Neck Cancer. Biomolecules.

[83] Tran N.H., Sais D., Tran N. (2024). Advances in Human Papillomavirus Detection and Molecular Understanding in Head and Neck Cancers: Implications for Clinical Management. J. Med. Virol..

[84] Chantre-Justino M., Alves G., Delmonico L. (2022). Clinical Applications of Liquid Biopsy in HPV-negative and HPV-positive Head and Neck Squamous Cell Carcinoma: Advances and Challenges. Explor. Target. Antitumor Ther..

[85] Eberly H.W., Sciscent B.Y., Lorenz F.J., Rettig E.M., Goyal N. (2024). Current and Emerging Diagnostic, Prognostic, and Predictive Biomarkers in Head and Neck Cancer. Biomedicines.

[86] Chen A.M. (2023). De-Escalation Treatment for Human Papillomavirus–Related Oropharyngeal Cancer: Questions for Practical Consideration. Oncology.

[87] Reid P., Marcu L.G., Olver I., Moghaddasi L., Staudacher A.H., Bezak E. (2019). Diversity of Cancer Stem Cells in Head and Neck Carcinomas: The Role of HPV in Cancer Stem Cell Heterogeneity, Plasticity and Treatment Response. Radiother. Oncol..

[88] Keck M.K., Zuo Z., Khattri A., Stricker T.P., Brown C.D., Imanguli M., Rieke D., Endhardt K., Fang P., Brägelmann J. (2015). Integrative Analysis of Head and Neck Cancer Identifies Two Biologically Distinct HPV and Three Non-HPV Subtypes. Clin. Cancer Res..

[89] Schindele A., Holm A., Nylander K., Allard A., Olofsson K. (2022). Mapping Human Papillomavirus, Epstein–Barr Virus, Cytomegalovirus, Adenovirus, and P16 in Laryngeal Cancer. Discov. Oncol..

[90] Vojtechova Z., Sabol I., Salakova M., Smahelova J., Zavadil J., Turek L., Grega M., Klozar J., Prochazka B., Tachezy R. (2016). Comparison of the MiRNA Profiles in HPV-Positive and HPV-Negative Tonsillar Tumors and a Model System of Human Keratinocyte Clones. BMC Cancer.

[91] Sharma S., Munger K. (2020). The Role of Long Noncoding RNAs in Human Papillomavirus-Associated Pathogenesis. Pathogens.

[92] Jiang M., Liu F., Yang A.-G., Wang W., Zhang R. (2022). The Role of Long Non-Coding RNAs in the Pathogenesis of Head and Neck Squamous Cell Carcinoma. Mol. Ther. Oncolytics.

[93] Ludwig S., Sharma P., Wise P., Sposto R., Hollingshead D., Lamb J., Lang S., Fabbri M., Whiteside T.L. (2020). MRNA and MiRNA Profiles of Exosomes from Cultured Tumor Cells Reveal Biomarkers Specific for HPV16-Positive and HPV16-Negative Head and Neck Cancer. Int. J. Mol. Sci..

[94] Sannigrahi M.K., Sharma R., Singh V., Panda N.K., Rattan V., Khullar M. (2018). DNA Methylation Regulated MicroRNAs in HPV-16-Induced Head and Neck Squamous Cell Carcinoma (HNSCC). Mol. Cell Biochem..

[95] Sun Y., Wang Z., Qiu S., Wang R. (2021). Therapeutic Strategies of Different HPV Status in Head and Neck Squamous Cell Carcinoma. Int. J. Biol. Sci..

[96] Lechner M., Liu J., Masterson L., Fenton T.R. (2022). HPV-Associated Oropharyngeal Cancer: Epidemiology, Molecular Biology and Clinical Management. Nat. Rev. Clin. Oncol..

[97] García-Anaya M., Segado-Guillot S., Cabrera-Rodríguez J., Toledo-Serrano M., Medina-Carmona J., Gómez-Millán J. (2023). Dose and Volume De-Escalation of Radiotherapy in Head and Neck Cancer. Crit. Rev. Oncol. Hematol..

[98] Rosenberg A.J., Vokes E.E. (2021). Optimizing Treatment De-Escalation in Head and Neck Cancer: Current and Future Perspectives. Oncologist.

[99] Gillison M.L., Trotti A.M., Harris J., Eisbruch A., Harari P.M., Adelstein D.J., Jordan R.C.K., Zhao W., Sturgis E.M., Burtness B. (2019). Radiotherapy plus Cetuximab or Cisplatin in Human Papillomavirus-Positive Oropharyngeal Cancer (NRG Oncology RTOG 1016): A Randomised, Multicentre, Non-Inferiority Trial. Lancet.

[100] Mehanna H., Robinson M., Hartley A., Kong A., Foran B., Fulton-Lieuw T., Dalby M., Mistry P., Sen M., O’Toole L. (2019). Radiotherapy plus Cisplatin or Cetuximab in Low-Risk Human Papillomavirus-Positive Oropharyngeal Cancer (De-ESCALaTE HPV): An Open-Label Randomised Controlled Phase 3 Trial. Lancet.

[101] Yom S.S., Torres-Saavedra P., Caudell J.J., Waldron J.N., Gillison M.L., Xia P., Truong M.T., Kong C., Jordan R., Subramaniam R.M. (2021). Reduced-Dose Radiation Therapy for HPV-Associated Oropharyngeal Carcinoma (NRG Oncology HN002). J. Clin. Oncol..

[102] Ferris R.L., Flamand Y., Weinstein G.S., Li S., Quon H., Mehra R., Garcia J.J., Chung C.H., Gillison M.L., Duvvuri U. (2022). Phase II Randomized Trial of Transoral Surgery and Low-Dose Intensity Modulated Radiation Therapy in Resectable P16+ Locally Advanced Oropharynx Cancer: An ECOG-ACRIN Cancer Research Group Trial (E3311). J. Clin. Oncol..

[103] Rahman M.d.T., Ghosh A.K., Khatun R.A., Hussain Q.M., Mollah M.d.N.U., Hussain S.M.d.A., Habib A.K.M.A., Hosen M.M.A., Rahim I.U. (2024). Comparison of Outcome of Concurrent Chemoradiotherapy and Sequential Chemoradiotherapy in Locally Advanced, Inoperable Squamous Cell Carcinoma of Head and Neck Region. Saudi J. Med. Pharm. Sci..

[104] Wong S.J., Torres-Saavedra P.A., Saba N.F., Shenouda G., Bumpous J.M., Wallace R.E., Chung C.H., El-Naggar A.K., Gwede C.K., Burtness B. (2023). Radiotherapy Plus Cisplatin With or Without Lapatinib for Non–Human Papillomavirus Head and Neck Carcinoma. JAMA Oncol..

[105] Lassen P., Huang S.H., Su J., Waldron J., Andersen M., Primdahl H., Johansen J., Kristensen C.A., Andersen E., Eriksen J.G. (2022). Treatment Outcomes and Survival Following Definitive (Chemo)Radiotherapy in HPV-positive Oropharynx Cancer: Large-scale Comparison of DAHANCA vs PMH Cohorts. Int. J. Cancer.

[106] Holcomb A.J., Herberg M., Strohl M., Ochoa E., Feng A.L., Abt N.B., Mokhtari T.E., Suresh K., McHugh C.I., Parikh A.S. (2021). Impact of Surgical Margins on Local Control in Patients Undergoing single-modality Transoral Robotic Surgery for HPV-related Oropharyngeal Squamous Cell Carcinoma. Head Neck.

[107] Goel S., Gunasekera D., Krishnan G., Krishnan S., Hodge J., Lizarondo L., Foreman A. (2025). Is Transoral Robotic Surgery Useful as a Salvage Technique in Head and Neck Cancers: A Systematic Review and Meta Analysis. Head Neck.

[108] Burtness B., Harrington K.J., Greil R., Soulières D., Tahara M., de Castro G., Psyrri A., Basté N., Neupane P., Bratland Å. (2019). Pembrolizumab Alone or with Chemotherapy versus Cetuximab with Chemotherapy for Recurrent or Metastatic Squamous Cell Carcinoma of the Head and Neck (KEYNOTE-048): A Randomised, Open-Label, Phase 3 Study. Lancet.

[109] Saba N.F., Pamulapati S., Patel B., Mody M., Strojan P., Takes R., Mäkitie A.A., Cohen O., Pace-Asciak P., Vermorken J.B. (2023). Novel Immunotherapeutic Approaches to Treating HPV-Related Head and Neck Cancer. Cancers.

[110] Powell S.F., Liu S.V., Sukari A., Chung C.H., Bauml J., Haddad R.I., Gause C.K., Niewood M., Gammage L.L., Brown H. (2015). KEYNOTE-055: A Phase II Trial of Single Agent Pembrolizumab in Patients (Pts) with Recurrent or Metastatic Head and Neck Squamous Cell Carcinoma (HNSCC) Who Have Failed Platinum and Cetuximab. J. Clin. Oncol..

[111] Muro K., Chung H.C., Shankaran V., Geva R., Catenacci D., Gupta S., Eder J.P., Golan T., Le D.T., Burtness B. (2016). Pembrolizumab for Patients with PD-L1-Positive Advanced Gastric Cancer (KEYNOTE-012): A Multicentre, Open-Label, Phase 1b Trial. Lancet Oncol..

[112] Uppaluri R., Haddad R.I., Tao Y., Le Tourneau C., Lee N.Y., Westra W., Chernock R., Tahara M., Harrington K.J., Klochikhin A.L. (2025). Neoadjuvant and Adjuvant Pembrolizumab in Locally Advanced Head and Neck Cancer. N. Engl. J. Med..

[113] Vallianou N.G., Evangelopoulos A., Kounatidis D., Panagopoulos F., Geladari E., Karampela I., Stratigou T., Dalamaga M. (2023). Immunotherapy in Head and Neck Cancer: Where Do We Stand?. Curr. Oncol. Rep..

[114] Julian R., Savani M., Bauman J.E. (2021). Immunotherapy Approaches in HPV-Associated Head and Neck Cancer. Cancers.

[115] Pereira D., Martins D., Mendes F. (2022). Immunotherapy in Head and Neck Cancer When, How, and Why?. Biomedicines.

[116] Shamseddine A.A., Burman B., Lee N.Y., Zamarin D., Riaz N. (2021). Tumor Immunity and Immunotherapy for HPV-Related Cancers. Cancer Discov..

[117] Even C., Harrington K.J., Massarelli E., Klein Hesselink M., Visscher S., Fury M.G., Sanders F., Laban S., Fayette J., Oliva M. (2024). Results of a Randomized, Double-Blind, Placebo-Controlled, Phase 2 Study (OpcemISA) of the Combination of ISA101b and Cemiplimab versus Cemiplimab for Recurrent/Metastatic (R/M) HPV16-Positive Oropharyngeal Cancer (OPC). J. Clin. Oncol..

[118] Nabell L., Ho A.L., Posner M.R., Neupane P., Naqash A.-R., Wong S., Niu J.J., Pearson A.T., Laux D., Adkins D.R. (2023). 921P HB-200 Arenavirus-Based Immunotherapy plus Pembrolizumab as a First-Line Treatment of Patients with Recurrent/Metastatic HPV16 Positive Head and Neck Cancer. Ann. Oncol..

[119] Yan S., Zhang X., Li F., Yang A., Li H., Hu W., Lin Q., Li X., Du M., Chen J. (2025). Neoadjuvant Sintilimab and Chemotherapy Followed by Transoral Surgery for HPV-Positive Resectable Oropharyngeal Cancer: A Single-Arm, Two-Centre, Phase 2 Trial. eClinicalMedicine.

[120] Rosenberg A.J., Juloori A., Izumchenko E., Cursio J., Gooi Z., Blair E.A., Lingen M.W., Cipriani N., Hasina R., Starus A. (2024). Neoadjuvant HPV16-Specific Arenavirus-Based Immunotherapy HB-200 plus Chemotherapy Followed by Response-Stratified de-Intensification in HPV16+ Oropharyngeal Cancer: TARGET-HPV. J. Clin. Oncol..

[121] Chung C.H., Li J., Steuer C.E., Bhateja P., Johnson M., Masannat J., Poole M.I., Song F., Hernandez-Prera J.C., Molina H. (2022). Phase II Multi-Institutional Clinical Trial Result of Concurrent Cetuximab and Nivolumab in Recurrent and/or Metastatic Head and Neck Squamous Cell Carcinoma. Clin. Cancer Res..

[122] Aguayo F., Perez-Dominguez F., Osorio J.C., Oliva C., Calaf G.M. (2023). PI3K/AKT/MTOR Signaling Pathway in HPV-Driven Head and Neck Carcinogenesis: Therapeutic Implications. Biology.

[123] Li Q., Tie Y., Alu A., Ma X., Shi H. (2023). Targeted Therapy for Head and Neck Cancer: Signaling Pathways and Clinical Studies. Signal Transduct. Target. Ther..

[124] LEE N.Y., Sherman E.J., Schöder H., Wray R., White C., Dunn L., Hung T., Pfister D.G., Ho A.L., McBride S.M. (2024). Intra-Treatment Hypoxia Directed Major Radiation de-Escalation as Definitive Treatment for Human Papillomavirus-Related Oropharyngeal Cancer. J. Clin. Oncol..

[125] Burtness B., Flamand Y., Quon H., Weinstein G.S., Mehra R., Garcia J.J., Kim S., O’Malley B.W., Ozer E., Ikpeazu C. (2025). Long-Term Follow-Up of E3311, an ECOG-ACRIN Cancer Research Group Phase II Trial of Transoral Surgery and Risk-Based Adjuvant Treatment in Human Papillomavirus–Initiated Oropharynx Cancer. J. Clin. Oncol..

[126] Frank S.J., Busse P., Rosenthal D.I., Hernandez M., Swanson D.M., Garden A.S., Sturgis E.M., Ferrarotto R., Gunn G.B., Patel S.H. (2024). Phase III Randomized Trial of Intensity-Modulated Proton Therapy (IMPT) versus Intensity-Modulated Photon Therapy (IMRT) for the Treatment of Head and Neck Oropharyngeal Carcinoma (OPC). J. Clin. Oncol..

[127] Hirayama S., Al-Inaya Y., Aye L., Bryan M.E., Das D., Mendel J., Naegele S., Faquin W.C., Sadow P., Fisch A.S. (2024). Prospective Validation of CtHPVDNA for Detection of Minimal Residual Disease and Prediction of Recurrence in Patients with HPV-Associated Head and Neck Cancer Treated with Surgery. J. Clin. Oncol..

[128] Wei Y., Zhao Z., Ma X. (2022). Description of CRISPR-Cas9 Development and Its Prospects in Human Papillomavirus-Driven Cancer Treatment. Front. Immunol..

[129] Zhen S., Lu J., Liu Y.-H., Chen W., Li X. (2020). Synergistic Antitumor Effect on Cervical Cancer by Rational Combination of PD1 Blockade and CRISPR-Cas9-Mediated HPV Knockout. Cancer Gene Ther..

[130] Chen Y., Jiang H., Wang T., He D., Tian R., Cui Z., Tian X., Gao Q., Ma X., Yang J. (2020). In Vitro and in Vivo Growth Inhibition of Human Cervical Cancer Cells via Human Papillomavirus E6/E7 MRNAs’ Cleavage by CRISPR/Cas13a System. Antivir. Res..

[131] Nagarsheth N.B., Norberg S.M., Sinkoe A.L., Adhikary S., Meyer T.J., Lack J.B., Warner A.C., Schweitzer C., Doran S.L., Korrapati S. (2021). TCR-Engineered T Cells Targeting E7 for Patients with Metastatic HPV-Associated Epithelial Cancers. Nat. Med..

[132] Wang X., Sandberg M.L., Martin A.D., Negri K.R., Gabrelow G.B., Nampe D.P., Wu M.-L., McElvain M.E., Toledo Warshaviak D., Lee W.-H. (2021). Potent, Selective CARs as Potential T-Cell Therapeutics for HPV-Positive Cancers. J. Immunother..

[133] Sharkey Ochoa I., O’Regan E., Toner M., Kay E., Faul P., O’Keane C., O’Connor R., Mullen D., Nur M., O’Murchu E. (2022). The Role of HPV in Determining Treatment, Survival, and Prognosis of Head and Neck Squamous Cell Carcinoma. Cancers.

[134] Šimić I., Božinović K., Milutin Gašperov N., Kordić M., Pešut E., Manojlović L., Grce M., Dediol E., Sabol I. (2023). Head and Neck Cancer Patients’ Survival According to HPV Status, MiRNA Profiling, and Tumour Features—A Cohort Study. Int. J. Mol. Sci..

[135] Lai Y.-H., Su C.-C., Wu S.-Y., Hsueh W.-T., Wu Y.-H., Chen H.H.W., Hsiao J.-R., Liu C.-H., Tsai Y.-S. (2022). Impact of Alcohol and Smoking on Outcomes of HPV-Related Oropharyngeal Cancer. J. Clin. Med..

[136] de Roest R.H., van der Heijden M., Wesseling F.W.R., de Ruiter E.J., Heymans M.W., Terhaard C., Vergeer M.R., Buter J., Devriese L.A., de Boer J.P. (2022). Disease Outcome and Associated Factors after Definitive Platinum Based Chemoradiotherapy for Advanced Stage HPV-Negative Head and Neck Cancer. Radiother. Oncol..

[137] Zhou J.Z., Jou J., Cohen E. (2021). Vaccine Strategies for Human Papillomavirus-Associated Head and Neck Cancers. Cancers.

[138] St Laurent J., Luckett R., Feldman S. (2018). HPV Vaccination and the Effects on Rates of HPV-Related Cancers. Curr. Probl. Cancer.

[139] Malagón T., Franco E.L., Tejada R., Vaccarella S. (2024). Epidemiology of HPV-Associated Cancers Past, Present and Future: Towards Prevention and Elimination. Nat. Rev. Clin. Oncol..

[140] Petca A., Borislavschi A., Zvanca M., Petca R.-C., Sandru F., Dumitrascu M. (2020). Non-Sexual HPV Transmission and Role of Vaccination for a Better Future (Review). Exp. Ther. Med..

[141] Du E.Y., Adjei Boakye E., Taylor D.B., Kuziez D., Rohde R.L., Pannu J.S., Simpson M.C., Patterson R.H., Varvares M.A., Osazuwa-Peters N. (2022). Medical Students’ Knowledge of HPV, HPV Vaccine, and HPV-Associated Head and Neck Cancer. Hum. Vaccin. Immunother..

[142] Porras C., Tsang S.H., Herrero R., Guillén D., Darragh T.M., Stoler M.H., Hildesheim A., Wagner S., Boland J., Lowy D.R. (2020). Efficacy of the Bivalent HPV Vaccine against HPV 16/18-Associated Precancer: Long-Term Follow-up Results from the Costa Rica Vaccine Trial. Lancet Oncol..

[143] Gribb J.P., Wheelock J.H., Park E.S. (2023). Human Papilloma Virus (HPV) and the Current State of Oropharyngeal Cancer Prevention and Treatment. Dela J. Public. Health.

[144] Roy V., Jung W., Linde C., Coates E., Ledgerwood J., Costner P., Yamshchikov G., Streeck H., Juelg B., Lauffenburger D.A. (2023). Differences in HPV-Specific Antibody Fc-Effector Functions Following Gardasil^®^ and Cervarix^®^ Vaccination. NPJ Vaccines.

[145] Smalley Rumfield C., Roller N., Pellom S.T., Schlom J., Jochems C. (2020). Therapeutic Vaccines for HPV-Associated Malignancies. Immunotargets Ther..

[146] Boilesen D.R., Nielsen K.N., Holst P.J. (2021). Novel Antigenic Targets of HPV Therapeutic Vaccines. Vaccines.

[147] Gardella B., Gritti A., Soleymaninejadian E., Pasquali M., Riemma G., La Verde M., Schettino M., Fortunato N., Torella M., Dominoni M. (2022). New Perspectives in Therapeutic Vaccines for HPV: A Critical Review. Medicina.

[148] Skolnik J.M., Morrow M.P. (2023). Vaccines for HPV-Associated Diseases. Mol. Asp. Med..

[149] Timbang M.R., Sim M.W., Bewley A.F., Farwell D.G., Mantravadi A., Moore M.G. (2019). HPV-Related Oropharyngeal Cancer: A Review on Burden of the Disease and Opportunities for Prevention and Early Detection. Hum. Vaccin. Immunother..

[150] Diana G., Corica C. (2021). Human Papilloma Virus Vaccine and Prevention of Head and Neck Cancer, What Is the Current Evidence?. Oral Oncol..

[151] Morand G.B., Cardona I., Cruz S.B.S.C., Mlynarek A.M., Hier M.P., Alaoui-Jamali M.A., da Silva S.D. (2022). Therapeutic Vaccines for HPV-Associated Oropharyngeal and Cervical Cancer: The Next De-Intensification Strategy?. Int. J. Mol. Sci..

[152] Tsentemeidou A., Fyrmpas G., Stavrakas M., Vlachtsis K., Sotiriou E., Poutoglidis A., Tsetsos N. (2021). Human Papillomavirus Vaccine to End Oropharyngeal Cancer. A Systematic Review and Meta-Analysis. Sex. Transm. Dis..

[153] Tsu V.D., LaMontagne D.S., Atuhebwe P., Bloem P.N., Ndiaye C. (2021). National Implementation of HPV Vaccination Programs in Low-Resource Countries: Lessons, Challenges, and Future Prospects. Prev. Med..

[154] Wang H., Jiang Y., Wang Q., Lai Y., Holloway A. (2023). The Status and Challenges of HPV Vaccine Programme in China: An Exploration of the Related Policy Obstacles. BMJ Glob. Health.

[155] Karanja-Chege C.M. (2022). HPV Vaccination in Kenya: The Challenges Faced and Strategies to Increase Uptake. Front. Public Health.

[156] Casey R.M., Adrien N., Badiane O., Diallo A., Loko Roka J., Brennan T., Doshi R., Garon J., Loharikar A. (2022). National Introduction of HPV Vaccination in Senegal—Successes, Challenges, and Lessons Learned. Vaccine.

[157] Agadayi E., Karademir D., Karahan S. (2022). Knowledge, Attitudes and Behaviors of Women Who Have or Have Not Had Human Papillomavirus Vaccine in Turkey about the Virus and the Vaccine. J. Community Health.

[158] Özdemir J., Yücel M., Kızılkaya S., Yıldırım G., Özyiğit I.İ., Yuluğkural Z. (2022). HPV, HPV Vaccination Worldwide and Current Status of HPV Vaccination in Turkey: A Literature Review. Turk. Med. Stud. J..

[159] Waheed D.-N., Schiller J., Stanley M., Franco E.L., Poljak M., Kjaer S.K., del Pino M., van der Klis F., Schim van der Loeff M.F., Baay M. (2021). Human Papillomavirus Vaccination in Adults: Impact, Opportunities and Challenges—A Meeting Report. BMC Proc..

[160] Nogueira-Rodrigues A., Flores M.G., Macedo Neto A.O., Braga L.A.C., Vieira C.M., de Sousa-Lima R.M., de Andrade D.A.P., Machado K.K., Guimarães A.P.G. (2022). HPV Vaccination in Latin America: Coverage Status, Implementation Challenges and Strategies to Overcome It. Front. Oncol..

[161] Erbıyık H.I., Palalıoğlu R.M. (2021). HPV Infection, HPV Vaccines and Cervical Cancer Awareness: A Multi-Centric Survey Study in Istanbul, Turkey. Women Health.

[162] Dilley S., Miller K.M., Huh W.K. (2020). Human Papillomavirus Vaccination: Ongoing Challenges and Future Directions. Gynecol. Oncol..

[163] Osazuwa-Peters N., Graboyes E.M., Khariwala S.S. (2020). Expanding Indications for the Human Papillomavirus Vaccine. JAMA Otolaryngol.-Head Neck Surg..

[164] Taberna M., Mena M., Pavón M.A., Alemany L., Gillison M.L., Mesía R. (2017). Human Papillomavirus-Related Oropharyngeal Cancer. Ann. Oncol..

[165] Ekanayake Weeramange C., Tang K.D., Irwin D., Hartel G., Langton-Lockton J., Ladwa R., Kenny L., Taheri T., Whitfield B., Vasani S. (2024). Human Papillomavirus (HPV) DNA Methylation Changes in HPV-Associated Head and Neck Cancer. Carcinogenesis.

[166] Wookey V.B., Appiah A.K., Kallam A., Ernani V., Smith L.M., Ganti A.K. (2019). HPV Status and Survival in Non-Oropharyngeal Squamous Cell Carcinoma of the Head and Neck. Anticancer. Res..

[167] Mandić K., Milutin Gašperov N., Božinović K., Dediol E., Krasić J., Sinčić N., Grce M., Sabol I., Barešić A. (2024). Integrative Analysis in Head and Neck Cancer Reveals Distinct Role of MiRNome and Methylome as Tumour Epigenetic Drivers. Sci. Rep..

[168] Shomorony A., Puram S.V., Johnson D.N., Chi A.W., Faquin W.C., Deshpande V., Deschler D.G., Emerick K.S., Sadow P.M. (2019). A Non-Oropharyngeal Squamous Cell Carcinoma and the Pitfalls of HPV Testing: A Case Report. Otolaryngol. Case Rep..

[169] Saito S., Shibata H., Adkins D., Uppaluri R. (2022). Neoadjuvant Immunotherapy Strategies in HPV-Related Head-and-Neck Cancer. Curr. Otorhinolaryngol. Rep..

[170] Yang S.-M., Wu M., Han F.-Y., Sun Y.-M., Yang J.-Q., Liu H.-X. (2021). Role of HPV Status and PD-L1 Expression in Prognosis of Laryngeal Squamous Cell Carcinoma. Int. J. Clin. Exp. Pathol..

[171] Allegra E., Bianco M.R., Mignogna C., Caltabiano R., Grasso M., Puzzo L. (2021). Role of P16 Expression in the Prognosis of Patients With Laryngeal Cancer: A Single Retrospective Analysis. Cancer Control.

[172] King R.E., Bilger A., Rademacher J., Lambert P.F., Thibeault S.L. (2023). Preclinical Models of Laryngeal Papillomavirus Infection: A Scoping Review. Laryngoscope.

[173] Ji M., Lin L., Huang Q., Hu C., Zhang M. (2023). HPV16 Status Might Correlate to Increasing Tumor-Infiltrating Lymphocytes in Hypopharyngeal Cancer. Acta Otolaryngol..

[174] Burbure N., Handorf E., Ridge J.A., Bauman J., Liu J.C., Giri A., Galloway T.J. (2021). Prognostic Significance of Human Papillomavirus Status and Treatment Modality in Hypopharyngeal Cancer. Head Neck.

[175] Patel E.J., Oliver J.R., Jacobson A.S., Li Z., Hu K.S., Tam M., Vaezi A., Morris L.G.T., Givi B. (2022). Human Papillomavirus in Patients With Hypopharyngeal Squamous Cell Carcinoma. Otolaryngol.-Head Neck Surg..

[176] Hebsgaard M., Eriksen P., Ramberg I., von Buchwald C. (2023). Human Papillomavirus in Sinonasal Malignancies. Curr. Otorhinolaryngol. Rep..

[177] Sjöstedt S., von Buchwald C., Agander T.K., Aanaes K. (2021). Impact of Human Papillomavirus in Sinonasal Cancer—A Systematic Review. Acta Oncol..

[178] Abi-Saab T., Lozar T., Chen Y., Tannenbaum A.P., Geye H., Yu M., Weisman P., Harari P.M., Kimple R.J., Lambert P.F. (2024). Morphologic Spectrum of HPV-Associated Sinonasal Carcinomas. Head Neck Pathol..

[179] Lauritzen B.B., Sjöstedt S., Jensen J.M., Kiss K., von Buchwald C. (2023). Unusual Cases of Sinonasal Malignancies: A Letter to the Editor on HPV-Positive Sinonasal Squamous Cell Carcinomas. Acta Oncol..

[180] Zhao B.Y., Hirayama S., Goss D., Zhao Y., Faden D.L. (2024). Human Papillomavirus-Associated Nasopharyngeal Carcinoma: A Systematic Review and Meta-Analysis. Oral Oncol..

[181] Hung S.-H., Yang T.-H., Lee H.-C., Lin H.-C., Chen C.-S. (2025). Association of Salivary Gland Cancer with Human Papillomavirus Infections. Eur. Arch. Oto-Rhino-Laryngol..

[182] Mohamed F.E., Aldayem L.N., Hemaida M.A., Siddig O., Osman Z.H., Shafig I.R., Salih M.A.M., Muneer M.S., Hassan R., Ahmed E.S. (2021). Molecular Detection of Human Papillomavirus-16 among Sudanese Patients Diagnosed with Squamous Cell Carcinoma and Salivary Gland Carcinoma. BMC Res. Notes.

[183] Costin C.A., Chifu M.B., Pricope D.L., Grigoraş A., Balan R.A., Amălinei C. (2024). Are HPV Oncogenic Viruses Involved in Salivary Glands Tumorigenesis?. Rom. J. Morphol. Embryol..

